# Amberlite-15 promoted an unprecedented aza Michael rearrangement for one pot synthesis of dihydroquinazolinone compounds†

**DOI:** 10.1039/c8ra03308k

**Published:** 2018-06-19

**Authors:** V. Narayana Murthy, Satish P. Nikumbh, Krishnaji Tadiparthi, M. V. Madhubabu, Subba Rao Jammula, L. Vaikunta Rao, Akula Raghunadh

**Affiliations:** Technology Development Centre, Custom Pharmaceutical Services, Dr Reddy's Laboratories Ltd Hyderabad 500049 India raghunadha@drreddys.com; Department of Chemistry, GIS, Gitam University Visakhapatnam 530045 India; Department of Chemistry, Christ University Hosur road Bangalore 560029 India

## Abstract

A new one pot multicomponent annulation strategy for the synthesis of various dihydroquinazolinone compounds has been developed using Amberlite-15 as a catalyst, giving good to moderate yields. In this reaction the substrate scope for amines and aldehydes was also investigated. The reaction has been checked on a large scale and the possible reaction mechanism has also been proposed.

## Abstract 2

A new one pot multicomponent annulation strategy for the synthesis of various dihydroquinazolinone compounds has been developed using Amberlite-15 as a catalyst, giving good to moderate yields.

## Graphical abstract

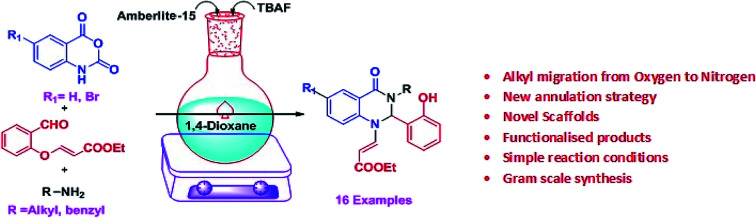

Functionalized dihydroquinazolinone frameworks are present in many biologically active molecules like quinethazone (A), fenquizone (D), benzouracil (B), evodiamine (C), NC1 substance1D19143 (E) and proquazone (F) (Fig. 1). Among the various dihydroquinazolinone compounds, some of them show various potential therapeutic effects, such as antidiabetic,^1^ anticancer,^2^ antihypertension,^3^ anticonvulsant,^4^ antibacterial,^5^ anti-inflammatory,^6^ and antianxietic activities.^7^ Moreover these compounds have also been used as antihistamine, antidepressant and vasodilating agents. Owing to their application in the medicinal and agro industries,^8,9^ many researchers around the world have put substantial efforts into the preparation of these compounds by various synthetic approaches.

**Fig. 1:**
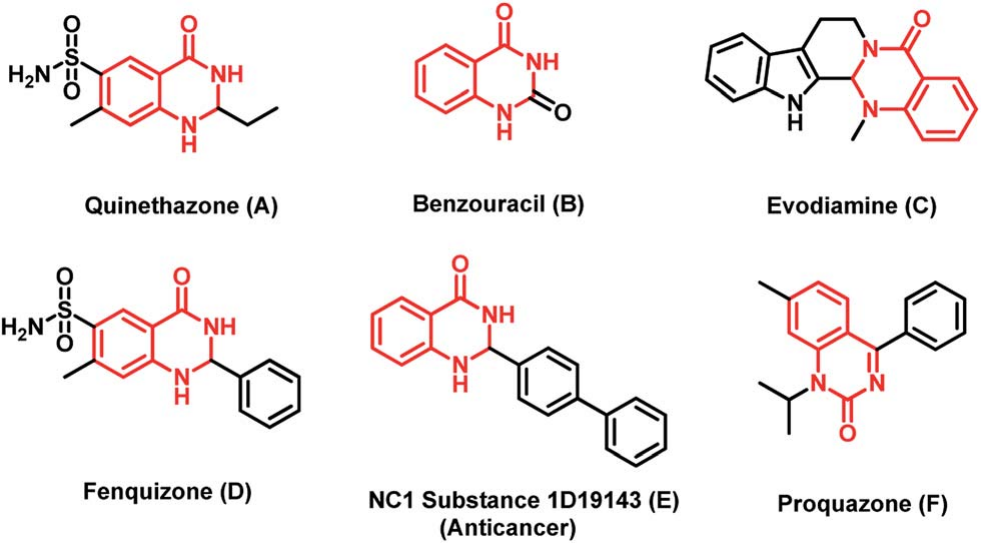
Few examples of natural and biologically active molecules with dihydroquinazolinone unit.

Indeed from the last few years various synthetic methods have been reported for the construction of various dihydroquinazolinones derivatives (Scheme 1).^10,11^ Bunce *et al.* reported the synthesis of substituted quinazolinones using a dissolving metal reduction–condensative cyclization strategy,^12^ Pal *et al.* synthesized 6,6*a*-dihydroisoindolo[2,1-*a*]quinazoline-5,11-dione derivatives by employing a three component reaction of isatoic anhydride, an amine and 2-formyl benzoic acid, using montmorillonite K10 as the catalyst,^13^ and the Sashidhara team also reported the synthesis of dihydroisoindolo[2,1-*a*]quinazoline-5,11-dione by using acetic acid.^14^ However among all the methods, the investigation of a practical and efficient multicomponent reaction for the synthesis of isoquinoline units with different nucleophiles has attracted many researchers because of their broad functional groups and simple construction of molecular architectures. Our group previously reported numerous protocols for the synthesis of quinazolinone based biologically active natural products and their derivatives. In continuation of our earlier efforts^15^ for the development of novel synthetic methods, herein we would like to report a new elegant one pot strategy for the construction of novel functionalized dihydroquinazolinones by the condensation of isatoic anhydride, amine and aldehyde.

**Scheme 1:**
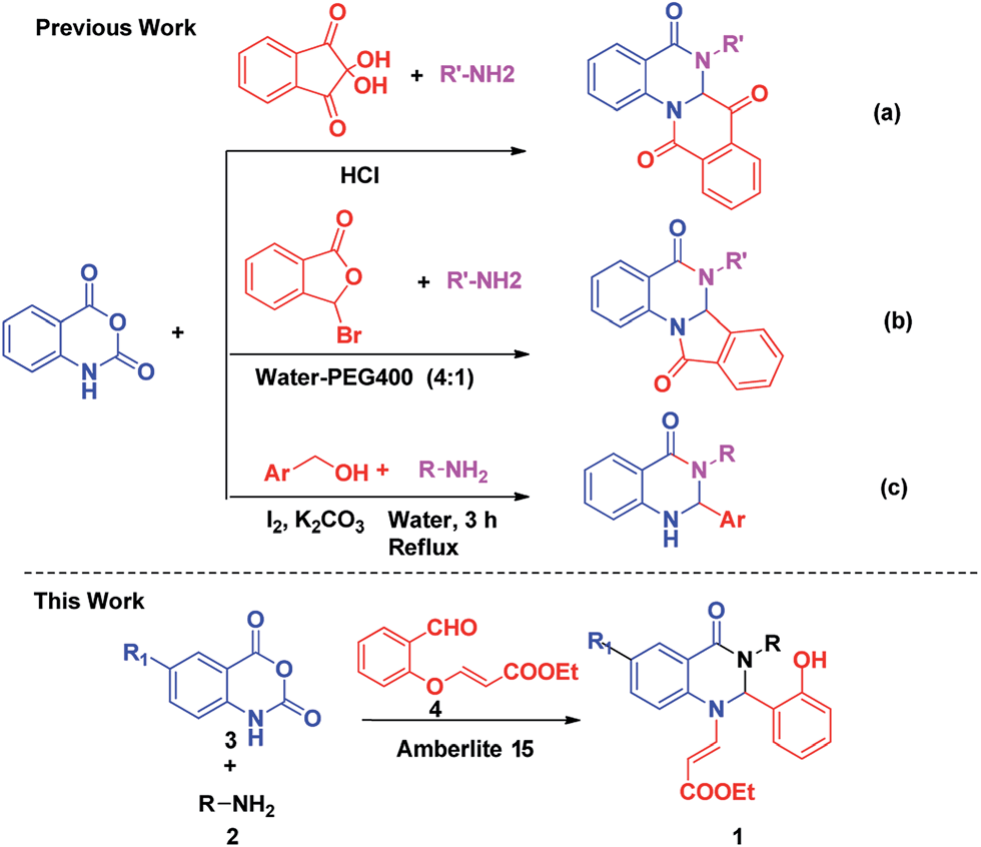
Various pathways for the construction of different dihydroquinazolinones moieties from isatoic anhydride.

We commenced our investigation with the model substrate by taking isatoic anhydride (3), butyl amine and aldehyde (4) as depicted in Table 1. In the first step of optimization, the reaction was screened with various catalysts like Wang resin, glacial acetic acid, BF_3_·OEt_2_, TiCl_4_, FeCl_3_ and Amberlite-15 in the presence of 1,4-dioxane as a solvent. Gratifyingly by using Amberlite-15 as a catalyst, we found a new functionalized dihydroquinazolinones compound. But using the other acid catalysts, the product with one double bond compound 13 was obtained as depicted in Fig. 2 along with the product 1. To optimize the molar ratio of the Amberlite-15 catalyst, initially we screened the reaction with 0.05 (w/w%) of Amberlite-15 and reaction was completed within 7–8 h and whereas with 0.10 (w/w%) of the catalyst, the reaction was completed within 3–4 h. In the next stage, the reaction was investigated in different solvents like DMSO, DMF, 1,4-dioxane, ethanol, methanol, THF, toluene and acetonitrile. Among all the solvents, the reaction was not progressed well with polar protic solvents like ethanol and methanol, whereas in polar aprotic solvents DMSO and DMF nearly 30% of the product formation was observed. Finally, we found that the reaction providing promising yields in 1,4-dioxane as solvent (Table 1). Indeed, the reaction rate is slow at room temperature and is fast under reflux conditions. Further the reaction was also checked in 10 and 20 vol but there is no difference in terms of yield and purity. The aza Michael addition step was attempted with 1 eq. of TBAF but only 15% of the product was observed whereas with 2 equivalent of TBAF, 60% of the product was observed whereas with3 equivalent of TBAF the reaction was completed.

**Table 1:** Screening of solvents using Amberlite-15 as catalyst

S. no.	Solvent	Isolated yield (%)
1	DMSO	28
2	DMF	30
3	1,4-Dioxane	64
4	Methanol	Trace
5	Ethanol	Trace
6	THF	48
7	Toluene	40
8	Acetonitrile	53

**Fig. 2:**
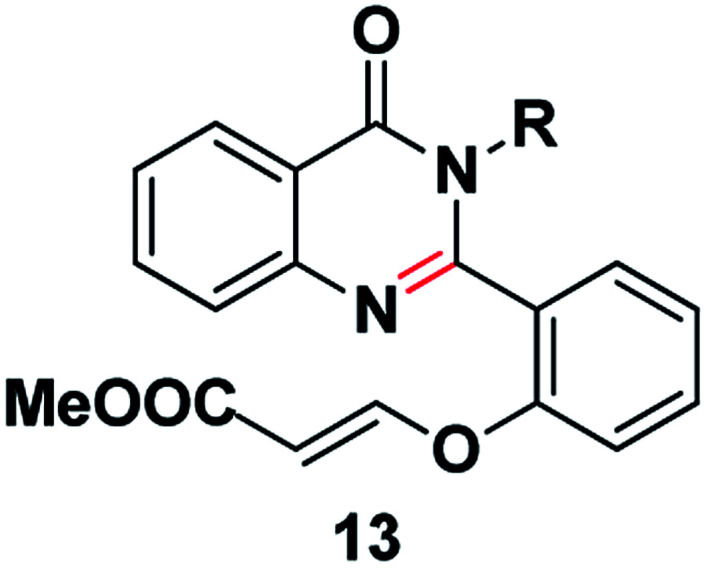
The product with other acid catalysts.

After optimizing the reaction conditions, we extended our studies for substrate scope of amines, aldehydes with isatoic anhydride and bromoisatoic anhydride. The substrate scope with various aliphatic, cyclic and aryl amines were studied and to our delight there is no abnormality in terms of yield and purity. This clearly states that all the substrates are well tolerated under these conditions to get the reasonable good yields (Table 2). Furthermore in order to elaborate the synthetic utility of this methodology we have extended our studies by performing a reaction on gram scale (Scheme 2) and gratefully we could replicate the yield and purity which clearly indicating that the reaction can be carried out on large scale.

**Table 2:** Synthesis of various dihydroquinazolinones derivatives^a^

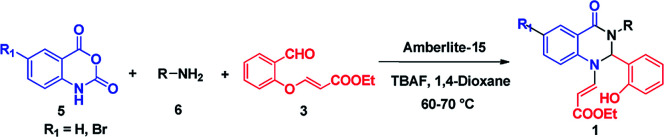
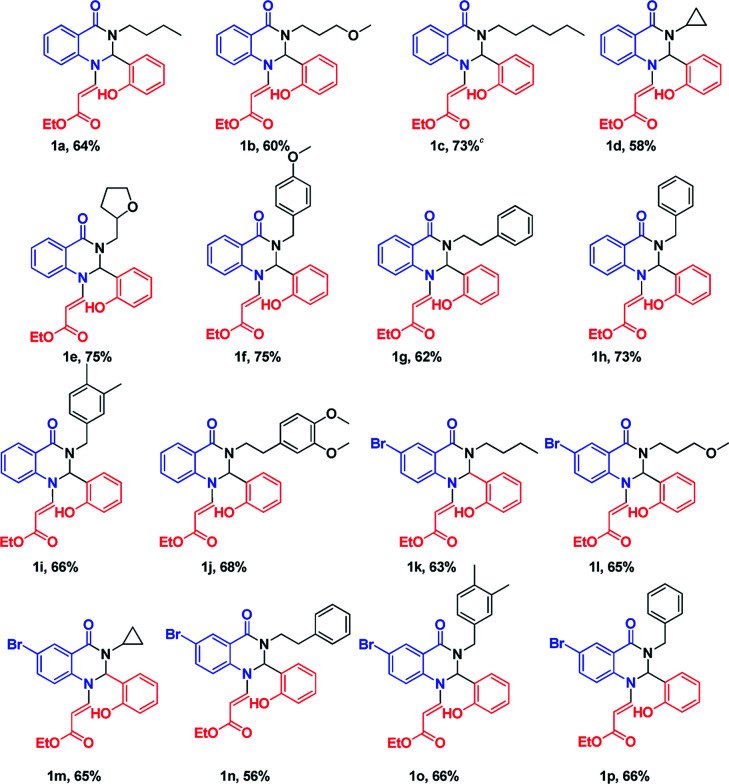

a Reaction conditions: isatoic anhydride (1 mmol), amine (1 mmol), aldehyde (1 mmol), Amberlite-15 (0.1 w/w%), 1,4-dioxane (5 mL).

**Scheme 2:**
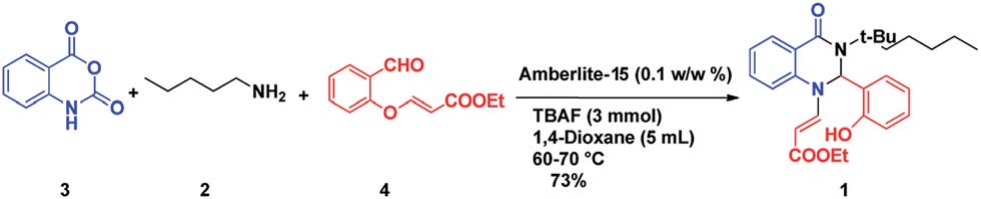
Gram scale experiment.

In order to gain insight into the mechanism, few control experiments have also been carried out. In the first experiment, the reaction with isatoic anhydride and amine without the aldehyde was carried out which provided the ring opening of the anhydride to get the anthranilide. In the second experiment, aldehyde with amine was carried out and the corresponding imine was obtained. In the third experiment, isatoic anhydride with aldehyde was carried out in the presence of the catalyst but the starting materials were intact (Scheme 3).

**Scheme 3:**
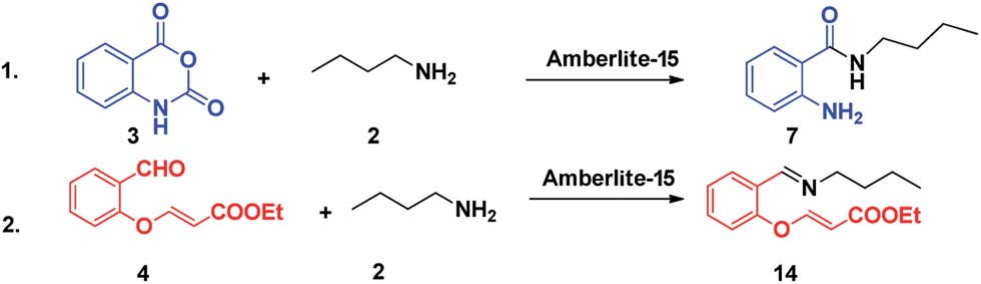
Control experiments.

From the above experimental data and literature reports, we have postulated a new possible reaction mechanism (Scheme 4). In addition to the spectroscopic evidence, the chemical structure of dihydroquinazolinone 1 was further confirmed from the single crystal X-ray diffraction analysis (Fig. 3).

**Scheme 4:**
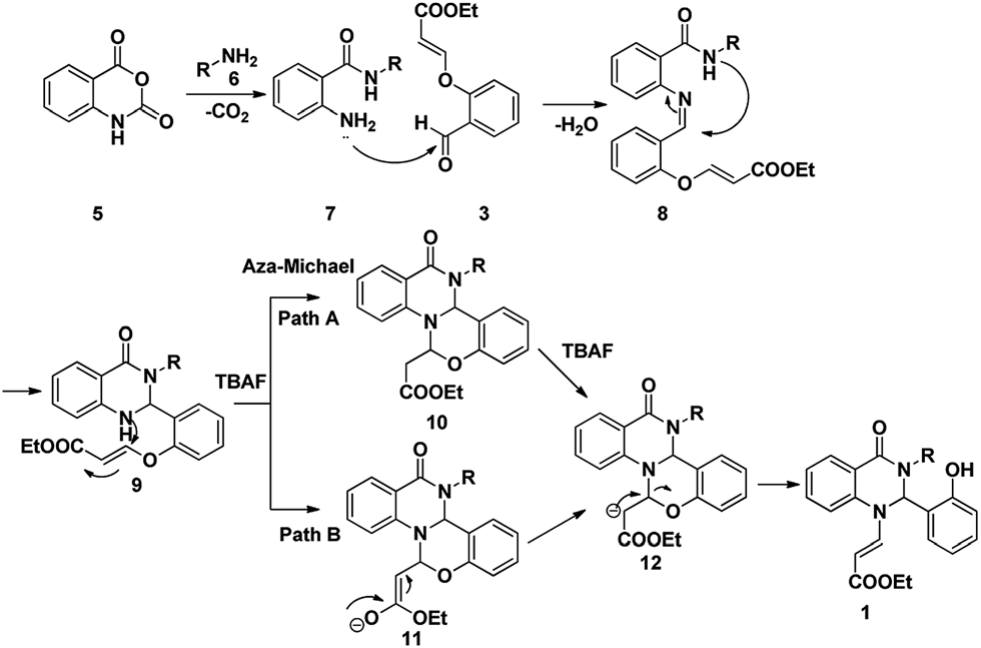
Plausible mechanism for the formation of dihydroquinazolinones

**Fig. 3:**
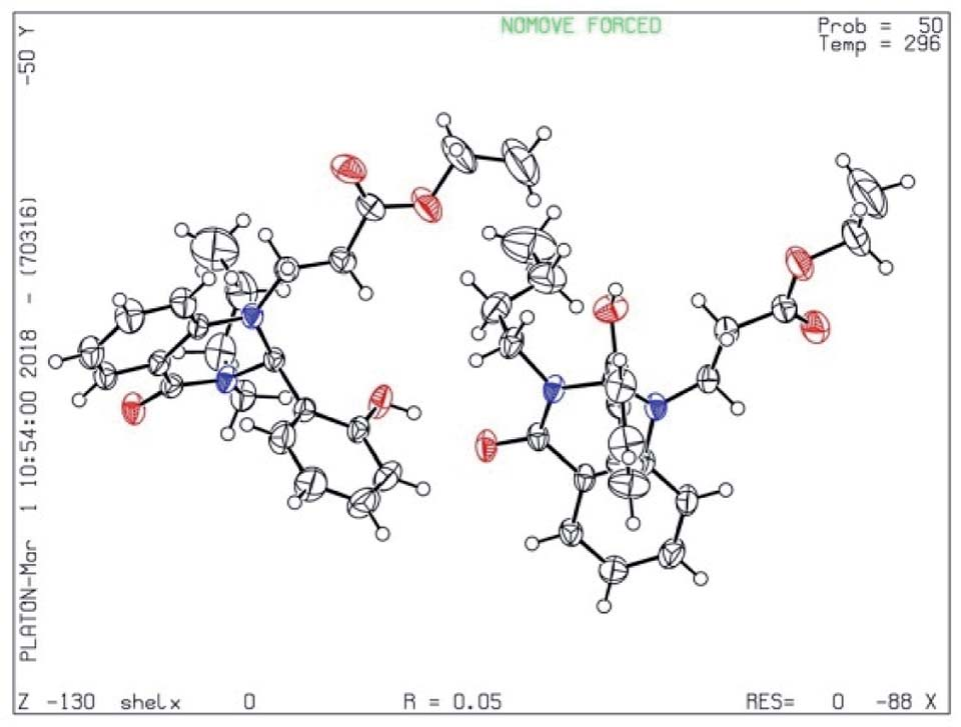
X-ray crystal structure of 1a (ORTEP diagram).

The Scheme 4 describes a plausible mechanism for the three component reaction leading to the compound 1. The nucleophilic attack of primary amine on carbonyl group of isatoic anhydride followed by ring opening and subsequent decarboxylation will yield to compound 7. Nucleophilic attack of amine to the aldehyde will yield imine intermediate 8; which on subsequent cyclization lead to the formation of 9. Nucleophilic attack of amine on the double bond *via* aza Michael reaction will yield the tetracyclic intermediate 10 or 11 which on further rearrangement leads to the formation of dihydroquinazolinones 1.

## Conclusion

In summary we have demonstrated the synthesis of novel functionalized dihydroquinazolinones using Amberlite-15 as a catalyst. It is a one pot multicomponent annulation strategy with broad substrate scope and the products obtained were in moderate to good yield. Moreover, the reaction is atom economic, metal-free and mild reaction conditions. Furthermore, these reactions are scalable and simple workup procedures. Further investigation of these types of model substrates is underway in our laboratory.

## Conflicts of interest

We confirm that this manuscript has not been published elsewhere and is not under consideration by any other journal. All of the authors agreed with submission to RSC Advance. We have no conflicts of interest to declare.

## Supplementary Material

RA-008-C8RA03308K-s001

RA-008-C8RA03308K-s002
