# Supplementary material for: Amberlite-15 promoted an unprecedented aza Michael rearrangement for one pot synthesis of dihydroquinazolinone compounds
Source: RSC Adv. 2018 Jun 19;8(40):22331–4. doi: 10.1039/c8ra03308k (PMC9092434; doi:10.1039/c8ra03308k)
Supplement: RA-008-C8RA03308K-s001 [file RA-008-C8RA03308K-s001.pdf]

Electronic Supplementary Material (ESI) for RSC Advances.
This journal is © The Royal Society of Chemistry 2018

Supporting Information

Amberlite-15 promoted an unprecedented aza Michael rearrangement for one pot synthesis of dihydroquinazolinone compounds

V. Narayana Murthy,[a,b] Satish P Nikumbh,[a] Krishnaji Tadiparthi,[c] M. V. Madhubabu,[a] Subba Rao. Jammula,[a] L. Vaikunta Rao,[b*] Akula Raghunadh[a*]

[a]*Technology Development Centre, Custom Pharmaceutical Services, Dr. Reddy's Laboratories Ltd, Hyderabad 500049, INDIA*

[b]*Department of Chemistry, GIS, Gitam University, Visakhapatnam 530045, INDIA*

[c]*Department of Chemistry, Christ University, Hosur road, Bangalore 560029, INDIA*

E-mail: raghunadha@drreddys.com

Section A: **General Information 2**

Section B: **Experimental Procedure.. ..3**

Section C: **^{1}H NMR, ^{13}C NMR and HRMS data 4**

Section D: **X-ray crystal structure and data of compound 1a 15**

Section A:

General methods: Unless stated otherwise, reactions were performed under nitrogen atmosphere using oven dried glassware. Reactions were monitored by thin layer chromatography (TLC) on silica gel plates (60 F254), visualizing with ultraviolet light or iodine spray. Flash chromatography was performed on silica gel (230-400 mesh) using distilled hexane, ethyl acetate, dichloromethane. ^{1}H NMR and ^{13}C NMR spectra were determined in $CDCl_3$solution by using 400 or 100 MHz spectrometers, respectively. Proton chemical shifts (δ) are relative to tetramethylsilane (TMS, δ = 0.00) as internal standard and expressed in ppm. Spin multiplicities are given as s (singlet), d (doublet), t (triplet) and m (multiplet) as well as b (broad). Coupling constants (*J*) are given in hertz. Melting points were determined using melting point B-540 apparatus and are uncorrected. HRMS was determined using waters LCT premier XETOF ARE-047 apparatus.

Section B:

Synthesis of 3-Substituted-2-(2-hydroxy-2-phenylvinyl)-2,3-dihydroquinazolin-4(1*H*)-one derivatives (1a-p)

General Procedure: To a solution of isatoic anhydride (1 mmol) in 1,4-dioxane (5 mL), amine (1 mmol) was added and the reaction mass was stirred for 3-4 h under reflux and the aldehyde (1 mmol) followed by amberlite-50 resin (0.10% w/w) were added sequentially and stirred under reflux another 3-4 h. the reaction mass was cooled to room temperature and TBAF (3 mmol) in THF was added and stirred at 60-70 °C for 2-3 hrs. After completion of the reaction, the solvent was evaporated and the crude residue was purified by column chromatography using Ethyl acetate / hexane (3:7) as an eluent.

Section C

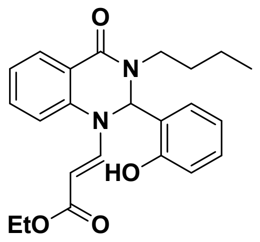

1a

(*E*)-Ethyl 3-(3-butyl-2-(2-hydroxyphenyl)-4-oxo-3,4-dihydroquinazolin-1(2*H*)-yl)acrylate (1a): White solid; Reaction was conducted in 5.0 grams scale and Output 8.3 grams with 64% Yield; m.p. 173-175 °C; ^{1}H NMR (400 MHz, DMSO-d_6): δ 10.32 (s, 1H, phenolic-OH), 7.93 (d, *J* = 7.7 Hz, 1H), 7.85 (d, *J* = 13.5 Hz, 1H), 7.54 (t, *J* = 7.4 Hz, 1H), 7.25-7.22 (m, 2H), 7.12 (t, *J* = 7.3 Hz, 1H), 6.86 (d, *J* = 7.9 Hz, 1H) 6.78 (d, *J* = 7.7 Hz, 1H), 6.63 (t, *J* = 7.6 Hz, 1H), 6.56 (s, 1H), 5.58 (d, *J* = 13.5 Hz, 1H), 4.11 (q, *J* = 7.1 Hz, 2H), 3.87-3.81 (m, 1H), 2.99-2.94 (m, 1H), 1.60-1.57 (m, 1H), 1.47-1.44 (m, 1H), 1.29-1.24 (m, 2H),1.22 (t, *J* = 8.8 Hz, 3H), 0.86 (t, *J* = 7.1 Hz, 3H);^{13}C NMR (100 MHz, DMSO-d_6):δ 167.5, 160.3, 154.6, 144.4, 140.2, 133.7, 130.2, 127.8, 126.0, 123.8, 123.1, 120.3, 119.3, 117.8, 115.7, 93.3, 67.5, 59.0, 43.9, 29.3, 19.4, 14.38,13.5; HRMS: m/z [M+1] calcd for $C_{23}H_{27}N_2O_4$: 395.1971; found: 395.1986.

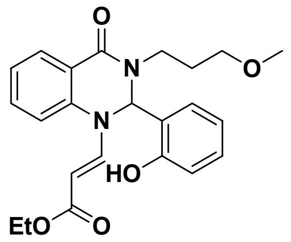

1b

(*E*)-Ethyl 3-(2-(2-hydroxyphenyl)-3-(3-methoxypropyl)-4-oxo-3,4-dihydroquinazolin-1(2*H*)-yl)acrylate (1b): White solid; Yield: 60% (0.755g from 0.500g isatoic anhydride); m.p. 159-161 °C; ^{1}H NMR (400 MHz, $CDCl_3$): δ 8.13 (d, *J* = 7.6 Hz, 1H), 7.95 (d, *J* = 14.0Hz, 1H), 7.75 (s,

1H), 7.44 (t, J = 6.8 Hz, 1H),7.21 (t, J = 7.6 Hz, 1H), 7.14 (t, J = 7.6 Hz, 1H),7.08 (d, J = 8.0 Hz, 1H), 6.95 (d, J = 7.6 Hz, 1H), 6.90 (d, J = 8.4 Hz, 1H), 6.68 (t, J = 7.2 Hz, 1H),6.61 (s, 1H), 5.68 (d, J = 13.2 Hz, 1H),4.26 (q, J = 6.8 Hz, 2H), 4.08-4.03 (m, 1H),3.53-3.42 (m, 2H), 3.34 (s, 3H), 3.16-3.09 (m, 1H), 2.07-1.93 (m, 1H), 1.93-1.83 (m, 1H), 1.29 (t, J = 7.6 Hz, 3H);^{13}C NMR (100 MHz, $CDCl_3$):δ 169.6, 161.9, 154.1, 145.2, 140.6, 133.6, 130.5, 128.5, 126.7, 124.1, 122.9, 120.7, 120.3, 117.8, 115.9, 93.9, 69.8, 68.4, 60.1, 58.6, 42.7, 27.7, 14.5; HRMS: m/z [M+1] calcd for $C_{23}H_{27}N_2O_4$: 395.1603; found: 395.1601.

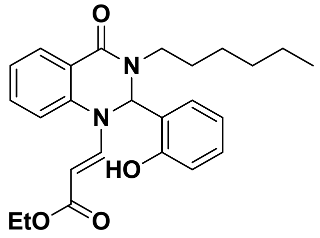

1c

(*E*)-Ethyl-3-(3-hexyl-2-(2-hydroxyphenyl)-4-oxo-3,4-dihydroquinazolin-1(2*H*)-yl)acrylate (1c): White solid; Yield: 73%(0.946g from 0.500g isatoic anhydride); m.p.: 161-163 °C; ^{1}H NMR (400 MHz, $CDCl_3$): δ 8.13 (d, J = 8.0 Hz, 1H), 7.94 (d, J = 13.6 Hz, 1H), 7.65 (s, 1H), 7.42 (t, J = 7.2 Hz, 1H), 7.21 (t, J = 7.2 Hz, 1H), 7.14 (t, J = 6.8 Hz, 1H),7.05 (d, J = 8.4 Hz, 1H), 6.96 (d, J = 7.6 Hz, 1H), 6.92 (d, J = 8.4 Hz, 1H), 6.68 (t, J = 7.6 Hz, 1H),6.55 (s, 1H), 5.69 (d, J = 13.6 Hz, 1H), 4.26 (q, J = 7.2 Hz, 2H), 4.08-4.01 (m, 1H), 2.98-2.91 (m, 1H),1.70-1.59 (m, 2H), 1.32 (t, J = 6.4 Hz, 3H), 1.28-1.20 (m, 6H), 0.84 (t, J = 6.8 Hz, 3H); ^{13}C NMR (100 MHz, $CDCl_3$):δ 169.7, 161.5, 154.1, 145.3, 140.6, 133.5, 130.5, 128.5, 126.8, 124.2, 122.8, 120.8, 120.3, 117.8, 115.8, 93.8, 67.6, 60.2, 45.0, 31.4, 27.6, 28.5, 22.5, 14.5, 13.9; HRMS: m/z [M+1] calcd for $C_{25}H_{31}N_2O_4$: 423.2284; found: 423.2283.

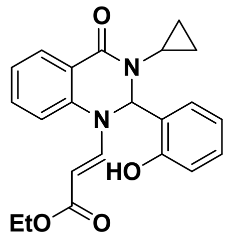

1d

(*E*)-Ethyl 3-(3-cyclopropyl-2-(2-hydroxyphenyl)-4-oxo-3,4-dihydroquinazolin-1(2*H*)-yl) acrylate (1d): Yellow solid; Yield: 58%(0.676g from 0.500g isatoic anhydride); mp: 218-220 °C; ^{1}H NMR (400 MHz, $CDCl_3$): δ 8.13 (d, *J* = 7.8 Hz, 1H), 7.89 (d, *J* = 13.2 Hz, 1H),7.56 (s, 1H), 7.34 (t, *J* = 7.3 Hz, 1H), 7.17-7.12 (m, 2H), 7.0-6.98 (m, 3H), 6.69 (t, *J* = 7.3 Hz, 1H), 6.53 (s, 1H), 5.72 (d, *J* = 13.7 Hz, 1H), 4.26 (q, *J* = 6.8 Hz, 2H), 2.69-2.66 (m, 1H),1.32 (t, *J* = 6.9 Hz, 3H), 1.09-1.06 (m, 1H), 0.92-0.90 (m, 1H), 0.81-0.76 (m, 1H), 0.70-0.67 (m, 1H); ^{13}C NMR (100 MHz, $CDCl_3$):δ 169.8, 164.1, 154.5, 145.7, 140.4, 133.7, 130.4, 128.5, 126.8, 124.1, 122.6, 121.0, 119.9, 118.2, 116.0, 93.7, 70.2, 60.2, 28.7,14.5, 9.2, 6.8; HRMS: m/z [M+1] calcd for $C_{22}H_{23}N_2O_4$: 379.1658; found: 379.1640.

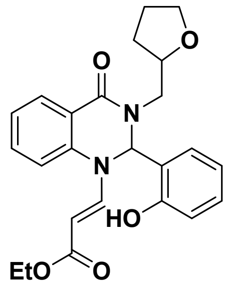

1e

(*E*)-Ethyl 3-(2-(2-hydroxyphenyl)-4-oxo-3-((tetrahydrofuran-2-yl)methyl)-3,4-dihydro quinazolin-1(2*H*)-yl)acrylate (1e): Yellow solid; Yield 75%(0.978g from 0.500g isatoic anhydride); m.p.: 195-197 °C; ^{1}H NMR (400 MHz, DMSO-d_6): δ 10.32 (s, 1H), 7.95 (d, *J* = 7.7 Hz, 1H), 7.83-7.78 (dd, J_1 = 7.4 Hz, J_2 = 6.1 Hz, 1H), 7.56-7.51 (m, 1H), 7.26-7.21 (m, 2H), 7.13-7.08 (m, 1H), 6.86-6.80 (m, 2H), 6.77-6.71 (m, 1H), 6.61 (t, *J* = 7.4 Hz,1H), 5.58-5.51 (m,

1H), 4.18-3.97 (m, 4H), 3.86-3.55 (m, 2H), 2.95-2.91 (m, 0.5H), 2.78-2.72 (m, 0.5H), 1.95-1.71 (m, 3H),1.65-1.45 (m, 1H), 1.19 (t, *J* = 7.1 Hz, 3H); ^{13}C NMR (100 MHz, DMSO-d_6):δ 167.6, 161.5, 155.0, 145.2, 133.8, 130.2, 127.8, 126.1 (2C), 124.1, 122.7, 119.3, 115.7 (2C), 93.3, 77.4, 67.3, 59.0 (2C), 47.8, 28.8, 25.46, 15.1 (2C); HRMS: m/z [M+1] calcd for $C_{24}H_{27}N_2O_5$: 423.1920; found: 423.1913.

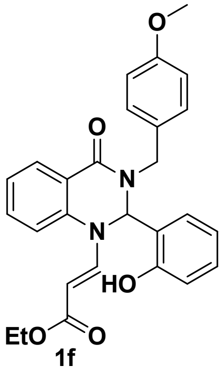

1f

(*E*)-Ethyl 3-(2-(2-hydroxyphenyl)-3-(4-methoxybenzyl)-4-oxo-3,4-dihydroquinazolin-1(2*H*)-yl)acrylate (1f): White solid; Yield: 75% (1.061g from 0.500g isatoic anhydride); m.p. 230-232 °C; ^{1}H NMR (400 MHz, DMSO-*d6*): δ 10.38 (s, 1H), 8.01 (d, *J* = 9.3 Hz, 1H), 7.75 (d, *J* = 13.5 Hz, 1H), 7.28-7.24 (m, 4H), 7.16-7.12 (m, 1H), 6.91-6.86 (m, 3H), 6.83 (d, *J* = 7.8 Hz, 1H), 6.65-6.61 (m, 1H), 6.45 (s, 1H), 5.34-5.24 (m, 2H), 4.07 (q, *J* = 7.1 Hz, 2H), 3.84(d, *J* = 4.9 Hz, 1H), 3.72 (s, 3H), 3.40-3.34 (m, 1H), 1.16 (t, *J* = 7.1 Hz, 3H); ^{13}C NMR (100 MHz, DMSO-d_6): δ 167.3, 160.2, 158.7, 154.5, 143.9, 140.4, 134.1, 130.4, 129.1 (2C), 128.5, 128.1, 126.1, 123.9, 122.6, 119.9, 117.7, 115.7, 113.9 (2C),93.5, 66.3, 64.9, 59.1, 55.3, 46.1, 14.3; HRMS: m/z [M+1] calcd for $C_{27}H_{26}N_2O_5$: 459.1920; found: 459.1897.

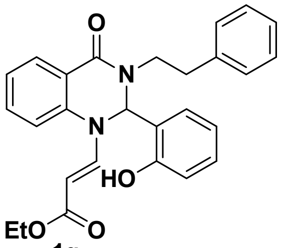

1g

(*E*)-Ethyl 3-(2-(2-hydroxyphenyl)-4-oxo-3-phenethyl-3,4-dihydroquinazolin-1(2*H*)-yl)acrylate (1g): White solid; Yield: 62%(0.846g from 0.500g isatoic anhydride); m.p.: 165-167 °C; ^{1}H NMR (400 MHz, $CDCl_3$): δ 8.15(d, *J* = 7.6 Hz, 1H), 7.75 (d, *J* = 6.8Hz, 1H), 7.46 (t, *J* = 6.8 Hz, 1H), 7.37 (s, 1H), 7.23-7.12 (m, 7H), 7.06 (d,*J* = 8.0 Hz, 1H), 6.94-6.88 (m, 2H), 6.67(t, *J* = 6.4 Hz,1H),6.25 (s, 1H), 5.39 (d, *J* = 13.6 Hz, 1H), 4.29-4.21 (m, 3H), 3.20-3.14 (m, 1H), 3.04-2.99 (m, 1H), 2.89-2.84 (m, 1H), 1.32(t, *J* = 7.2 Hz, 3H);^{13}C NMR (100 MHz, DMSO-d_6):δ 167.5, 160.3, 154.6, 144.1, 140.3, 138.6, 133.8, 130.3, 128.7 (2C), 128.4 (2C), 127.8, 126.3, 126.1, 123.8, 123.1, 120.1, 119.4, 117.8, 115.7, 93.5, 68.3, 59.01, 46.3, 33.5, 14.5; HRMS: m/z [M+1] calcd for $C_{27}H_{27}N_2O_4$: 443.1971; found: 443.1965.

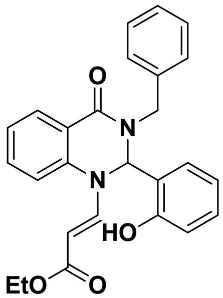

1h

(*E*)-Ethyl-3-(3-benzyl-2-(2-hydroxyphenyl)-4-oxo-3,4-dihydroquinazolin-1(2*H*)-yl)acrylate (1h): White solid; Yield: 73% (0.965g from 0.500g isatoic anhydride); m.p.: 279-281 °C; ^{1}H NMR (400 MHz, DMSO-d_6): δ 10.36 (s, 1H), 8.01 (d, *J* = 8.0 Hz, 1H), 7.77 (d, *J* = 13.6 Hz, 1H), 7.58 (t, *J* = 7.6 Hz, 1H), 7.35-7.25 (m, 7H), 7.14 (t, *J* = 8.4 Hz, 1H), 6.87-6.82 (m, 2H), 6.64(t, *J* = 7.6 Hz, 1H), 6.47 (s, 1H), 5.35-5.27 (m, 2H), 4.07-4.02 (q, *J* = 7.2 Hz, 2H), 3.97-3.93 (d, *J* = 15.6 Hz, 1H), 1.16 (t, *J* = 6.8 Hz, 3H); ^{13}C NMR (100 MHz, DMSO-d_6):δ 167.1, 160.3, 154.4,

143.8, 140.3, 136.6, 134.0, 130.3, 128.4 (2C), 127.9, 127.3 (3C), 125.9, 123.8, 122.4, 119.6, 119.3, 117.6, 115.6, 93.4, 66.7, 58.9, 46.7, 14.2; HRMS: m/z [M+1] calcd for $C_{26}H_{25}N_2O_4$: 429.1814; found: 429.1797.

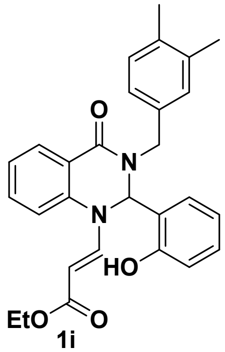

1i

(*E*)-Ethyl-3-(3-(3,4-dimethylbenzyl)-2-(2-hydroxyphenyl)-4-oxo-3,4-dihydroquinazolin-1(2*H*)-yl)acrylate (1i): White solid; Yield: 66%(0.930g from 0.500g isatoic anhydride); m.p. 210-212 °C; ^{1}H NMR (400 MHz, DMSO-d_6): δ 10.35 (s, 1H), 8.0 (d, *J* = 8.0 Hz, 1H), 7.73 (d, *J* = 13.6 Hz, 1H), 7.57 (t, *J* = 8.0 Hz, 1H), 7.28-7.25 (m, 2H), 7.16-7.01 (m, 4H), 7.86-7.81 (m, 2H), 6.64 (t, *J* = 7.6Hz, 1H), 6.42 (s, 1H), 5.31-5.27 (m, 2H), 4.07-4.0 (q, *J* = 7.2 Hz, 2H), 3.80 (d, *J* = 15.2 Hz, 1H), 2.17 (s, 3H), 2.15 (s, 3H), 1.15 (t, *J* = 7.2 Hz, 3H);^{13}C NMR (100 MHz, DMSO-d_6):δ 167.4, 160.4, 154.7, 144.2, 140.5, 136.4, 135.4, 134.1, 130.4, 129.7, 128.7, 128.1, 126.1, 125.0, 124.1, 122.5, 120.1, 119.4, 118.0, 115.8, 93.6, 66.6, 59.1, 46.3, 26.8, 19.4, 19.1, 14.4; HRMS: m/z [M+1] calcd for $C_{28}H_{29}N_2O_4$: 457.2127; found: 457.2100.

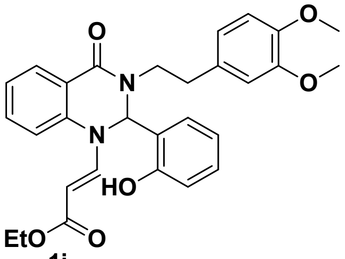

1j

(*E*)-Ethyl3-(3-(3,4-dimethoxyphenethyl)-2-(2-hydroxyphenyl)-4-oxo-3,4-dihydro quinazolin-1(2*H*)-yl)acrylate (1j): White solid; Yield: 68% (1.054g from 0.500g isatoic

anhydride); m.p. 230-232 °C; ^{1}H NMR (400 MHz, DMSO-d_6): δ 10.35 (s, 1H), 7.95 (d, J = 8.0 Hz, 1H), 7.72 (d, J = 13.6 Hz, 1H), 7.54 (t, J = 8.0 Hz, 1H), 7.24-7.21 (m, 2H), 7.11 (t, J = 8.0 Hz, 1H), 6.86 (d, J = 8.0 Hz, 1H), 6.80-6.75 (m, 3H), 6.71-6.68 (m, 1H), 6.61 (t, J = 7.6 Hz, 1H), 6.30 (s, 1H), 5.34 (d, J = 13.6 Hz, 1H), 4.10-4.03 (m, 3H), 3.67 (s, 6H), 3.16-3.08 (m, 1H), 2.89-2.82 (m, 1H), 2.73-2.67 (m, 1H), 1.20 (t, J = 7.2 Hz, 3H);^{13}C NMR (100 MHz, DMSO-d_6):δ 167.5, 160.4, 154.5, 148.6, 147.5, 143.8, 140.3, 133.9, 130.8, 130.3, 127.8, 126.1, 123.7, 123.1, 120.6, 120.0, 119.4, 117.6, 115.7, 112.5, 111.7, 93.5, 67.9, 59.0, 55.3 (2C), 46.2, 33.0, 14.4; HRMS: m/z [M+1] calcd for $C_{29}H_{31}N_2O_6$: 503.2182; found: 503.2186.

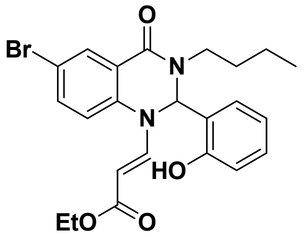

1k

(*E*)-Ethyl3-(6-bromo-3-butyl-2-(2-hydroxyphenyl)-4-oxo-3,4-dihydroquinazolin-1(2*H*)-yl)acrylate (1k): Yellow solid; Yield: 63% (0.619g from 0.500g isatoic anhydride);m.p. 186-188 °C; ^{1}H NMR (400 MHz, DMSO-d_6): δ 10.38 (s, 1H), 7.98 (d, J = 2.4 Hz, 1H), 7.83 (d, J = 13.2 Hz, 1H), 7.70-7.67 (dd, J_1 = 8.8 Hz, J_2 = 2.4 Hz, 1H),7.27 (d, J = 8.8 Hz, 1H), 7.14 (t, J = 8.4 Hz, 1H),6.86-6.81 (m, 2H), 6.67 (t, J = 7.6 Hz, 1H), 6.57 (s, 1H), 5.61 (d, J = 13.6 Hz, 1H),4.12 (q, J = 6.8 Hz, 2H), 3.84-3.77 (m, 1H), 3.03-2.96 (m, 1H), 1.62-1.41 (m, 2H),1.28-1.25 (m, 2H), 1.20 (t, J = 7.6 Hz, 3H), 0.86 (t, J = 7.6 Hz, 3H); ^{13}C NMR (100 MHz, DMSO-d_6):δ 167.4, 159.1, 154.6, 144.0, 139.5, 136.3, 130.4, 130.0, 126.2, 122.8, 121.9, 120.2, 119.4, 115.8, 115.6, 94.2, 67.8, 59.2, 44.2, 29.2, 19.4, 14.4,13.6; HRMS: m/z [M+1] calcd for $C_{23}H_{26}BrN_2O_4$: 473.1076; found: 473.1070.

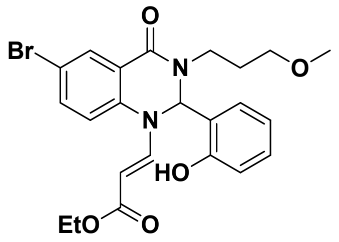

1l

(*E*)-Ethyl3-(6-bromo-2-(2-hydroxyphenyl)-3-(3-methoxypropyl)-4-oxo-3,4-dihydro quinazolin-1(2*H*)-yl)acrylate (1l): Yellow solid; Yield: 65% (0.660g from 0.500g isatoic anhydride); m.p. 189-191 °C; ^{1}H NMR (400 MHz, DMSO-d_6): δ 10.37 (s, 1H), 7.98 (d, *J* = 2.4 Hz, 1H), 7.83 (d, *J* = 13.6 Hz, 1H), 7.70-7.67 (dd, J_1 = 8.4 Hz, J_2 = 2.4 Hz, 1H),7.27 (d, *J* = 7.2 Hz, 1H), 7.14 (t, *J* = 7.2 Hz, 1H),6.85-6.79 (m, 2H), 6.67 (t, *J* = 7.2 Hz, 1H), 6.59 (s, 1H), 5.62 (d, *J* = 13.6 Hz, 1H),4.12 (q, *J* = 7.2 Hz, 2H), 3.88-3.81 (m, 1H),3.34 (s, 3H), 3.32-3.30 (m, 2H),3.07-2.99 (m, 1H),1.86-1.66 (m, 2H), 1.19 (t, *J* = 6.8 Hz, 3H);^{13}C NMR (100 MHz, DMSO-d_6):δ 167.4, 159.2, 154.7, 144.2, 139.5, 136.3, 130.4, 129.9, 126.2, 122.7, 122.0, 120.4, 119.4, 115.8, 115.6, 94.2, 69.3, 68.4, 59.1, 57.8, 42.5, 27.4, 14.4; HRMS: m/z [M+1] calcd for $C_{23}H_{26}BrN_2O_5$: 395.1971; found: 395.1986.

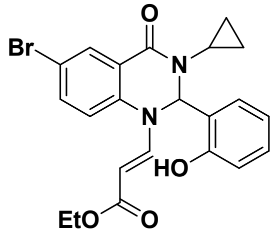

1m

(*E*)-Ethyl 3-(6-bromo-3-cyclopropyl-2-(2-hydroxyphenyl)-4-oxo-3,4-dihydroquinazolin-1(2*H*)-yl)acrylate (1m): Yellow solid; Yield: 65% (0.617g from 0.500g isatoic anhydride); m.p. 243-245 °C; ^{1}H NMR (400 MHz, DMSO-d_6): δ 10.35 (s, 1H), 7.99 (s, 1H), 7.81 (d, *J* = 13.6 Hz, 1H), 7.67 (d, *J* = 8.8 Hz, 1H), 7.25 (d, *J* = 8.8 Hz, 1H),7.13 (t, *J* = 7.6 Hz, 1H), 6.84 (t, *J* = 7.6 Hz, 2H), 6.67 (t, *J* = 7.2 Hz, 1H), 6.45 (s, 1H), 5.65 (d, *J* = 13.6 Hz, 1H), 4.11 (q, *J* = 6.8 Hz,

2H), 1.20 (t, J = 6.8 Hz, 3H), 1.09-1.06 (m, 1H), 0.95-0.91 (m, 1H), 0.72-0.71 (m, 3H);^{13}C NMR (100 MHz, $CDCl_3$):δ 167.5, 161.2, 154.7, 144.4, 139.2, 136.4, 130.4, 129.8, 126.2, 122.6, 122.3, 120.6, 119.3, 115.9, 115.7, 94.2, 69.8, 59.2, 28.2, 14.4, 8.7, 6.1; HRMS: m/z [M+1] calcd for $C_{22}H_{22}BrN_2O_4$: 457.0763; found: 457.0757.

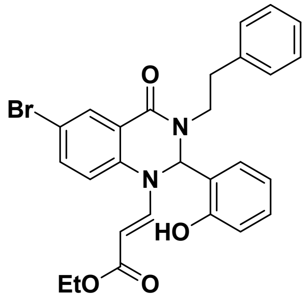

1n

(*E*)-Ethyl 3-(6-bromo-2-(2-hydroxyphenyl)-4-oxo-3-phenethyl-3,4-dihydroquinazolin-1(2*H*)-yl)acrylate (1n): Yellow solid; Yield: 56% (0.606g from 0.500g isatoic anhydride);m.p. 181-183 °C; ^{1}H NMR (400 MHz, DMSO-d_6): δ 10.35 (s, 1H), 7.99 (d, J = 2.4 Hz, 1H), 7.70-7.64 (m, 2H), 7.27-7.11 (m, 7H), 6.85-6.80 (m, 2H), 6.66 (t, J = 7.6 Hz, 1H), 6.35 (s, 1H), 5.37 (d, J = 13.6 Hz, 1H), 4.13 (q, J = 7.6 Hz, 2H), 4.03-3.99 (m, 1H), 3.22-3.15 (m, 1H), 2.96-2.88 (m, 1H), 2.78-2.67 (m, 1H), 1.22 (t, J = 7.2 Hz, 3H); ^{13}C NMR (100 MHz, DMSO-d_6):δ 167.2, 159.0, 154.6, 143.6, 139.6, 138.4, 136.3, 130.4, 129.9, 128.6 (2C), 128.4 (2C), 126.3 (2C), 122.7, 121.7, 120.2, 119.4, 115.7, 115.5, 94.3, 68.5, 59.1, 46.4, 33.3, 14.4; Mass: m/z [M+1] for $C_{27}H_{25}BrN_2O_4$: 521.40; found: 523.20.

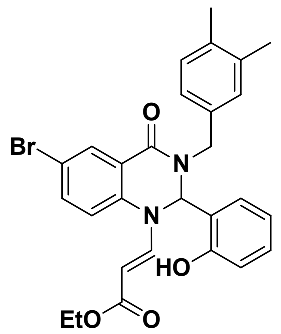

1o

(*E*)-Ethyl 3-(6-bromo-3-(3,4-dimethylbenzyl)-2-(2-hydroxyphenyl)-4-oxo-3,4-dihydro quinazolin-1(2*H*)-yl)acrylate (1o): Yellow solid; Yield: 66% (0.733g from 0.500g isatoic anhydride); m.p.: 210-212 °C; ^{1}H NMR (400 MHz, DMSO-d_6): δ 10.38 (s, 1H), 8.05 (d, *J* = 2.4 Hz, 1H), 7.72-7.68 (m, 2H), 7.27 (d, *J* = 8.8 Hz, 1H), 7.15 (t, *J* = 8.0 Hz, 1H), 7.09-7.05 (m, 2H), 7.02 (d, *J* = 7.6 Hz, 1H), 6.87 (d, *J* = 8.0 Hz, 2H), 6.68 (t, *J* = 8.0 Hz, 1H), 6.44 (s, 1H), 5.35 (d, *J* = 13.2 Hz, 1H), 5.27 (d, *J* = 15.2 Hz, 1H), 4.08 (q, *J* = 7.2 Hz, 2H), 3.84 (d, *J* = 15.2 Hz, 1H), 2.17 (s, 3H), 2.15 (s, 3H), 1.17 (t, *J* = 6.8 Hz, 3H);^{13}C NMR (100 MHz, DMSO-d_6):δ 167.1, 159.2, 154.7, 143.7, 139.7, 136.5, 136.4, 135.4, 133.6, 130.5, 130.2, 129.7, 128.7, 126.2, 125.0, 122.2, 121.6, 120.3, 119.5, 115.8, 115.7, 94.4, 66.7, 59.2, 46.5, 19.3, 19.0, 14.4; HRMS: m/z [M+1] calcd for $C_{28}H_{28}BrN_2O_4$: 535.1232; found: 535.1193.

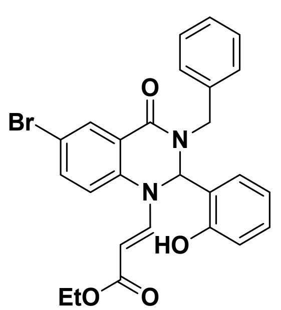

1p

(*E*)-Ethyl-3-(3-benzyl-6-bromo-2-(2-hydroxyphenyl)-4-oxo-3,4-dihydroquinazolin-1(2*H*)-yl)acrylate(1p): Yellow solid; Yield: 66% (0.695g from 0.500g isatoic anhydride); m.p.: 215-217 °C; ^{1}H NMR (400 MHz, DMSO-d_6): δ 10.39 (s, 1H), 8.04 (s, 1H), 7.75-7.71 (m, 2H), 7.33-7.27 (m, 5H), 7.17-7.13 (m, 1H), 6.87-6.84 (m, 2H), 6.67 (t, *J* = 6.9 Hz, 1H), 6.48 (s, 1H), 5.38

(d, J = 13.5Hz, 1H), 5.27-5.20 (m, 1H), 4.07-4.02 (q, J = 7.1 Hz, 2H), 4.00-3.96 (d, J = 15.3 Hz, 1H), 1.16 (t, J = 7.1 Hz, 3H); ^{13}C NMR (100 MHz, DMSO-d_6):δ 167.1, 159.2, 154.5, 143.5, 139.7, 136.6, 130.5, 130.1, 128.5 (2C), 127.6 (3C), 126.1, 122.2, 121.3, 120.1, 119.5, 115.8, 115.6, 94.3, 67.0, 59.1, 46.9, 26.8, 14.3; HRMS: m/z [M+1] calcd for $C_{26}H_{24}N_2O_4Br$: 507.0919; found: 507.0925.

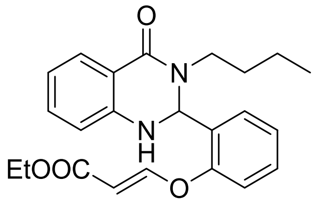

Intermediate

(*E*)-Ethyl 3-(2-(3-butyl-4-oxo-1,2,3,4-tetrahydroquinazolin-2-yl)phenoxy)acrylate (Intermediate): White solid; Yield: 78% (0.949g from 0.500g isatoic anhydride); m.p.: 179-181 °C; ^{1}H NMR (400 MHz, $CDCl_3$): δ 7.95 (d, J = 8.8 Hz, 1H), 7.80 (d, J = 12.2 Hz, 1H), 7.33 (t, J = 6.3 Hz, 1H), 7.24-7.17 (m, 2H), 7.12-7.07 (m, 2H), 6.81 (t, J = 6.8 Hz, 1H), 6.48 (d, J = 7.8 Hz, 1H), 5.98 (s, 1H), 5.67 (d, J = 12.2 Hz, 1H), 4.74 (s, 1H), 4.27 (m, 3H), 2.70-2.63 (m, 1H),1.67-1.61 (m, 2H),1.42-1.36 (m, 2H), 1.32 (t, J = 7.6 Hz, 3H), 0.92 (t, J = 7.4 Hz, 3H); ^{13}C NMR (100 MHz, DMSO-d_6):δ165.9, 162.1, 158.7, 152.5, 145.8, 133.1, 130.7, 130.3, 127.3, 127.1, 125.0, 118.0, 117.0, 114.4, 114.2, 102.5, 65.3, 59.6, 43.6, 29.3, 19.5, 14.2, 13.6; HRMS: m/z[M+1] calcd for $C_{23}H_{27}N_2O_4$: 395.1971; found: 395.1955.

Section D

X-ray crystal structure and data of compound 1a:

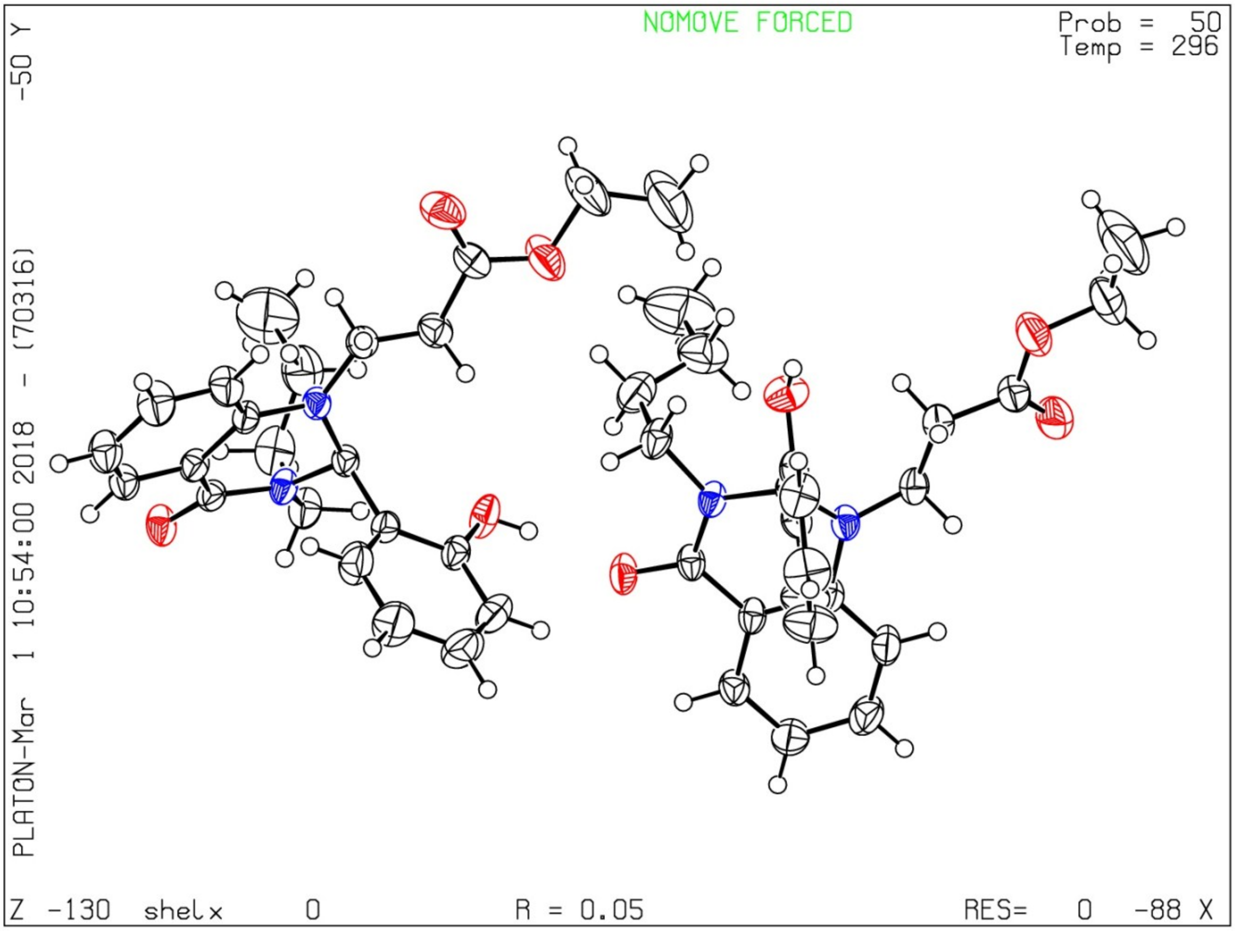

Fig. 1 ORTEP diagram of **1a**

Identification code	***shelx***
Empirical formula	*$C_{23} H_{27} N_2 O_4$*
Formula weight	*395.46*
Temperature	*296(2) K*
Wavelength	*0.71073 A*

Crystal system, space group	*?, 0*
Unit cell dimensions	*a = 21.7483(19) A alpha = 90 deg.*
	b = 9.2050(7) A beta = 123.852(7) deg.
	c = 13.1074(15) A gamma = 90 deg.
Volume	*2179.2(4) A^3*
Z, Calculated density	*4, 1.205 Mg/m^3*
Absorption coefficient	*0.083 mm^-1*
F(000)	*844*
Crystal size	*0.350 x 0.300 x 0.300 mm*
Theta range for data collection	*1.871 to 28.316 deg.*
Limiting indices	*-27<=h<=28, -12<=k<=12, -17<=l<=17*
Reflections collected / unique	*13371 / 8631 [R(int) = 0.0207]*
***Completeness to theta** = 25.242*	*99.7 %*
Absorption correction	*Semi-empirical from equivalents*
Max. and min. transmission	*0.976 and 0.972*
Refinement method	*Full-matrix least-squares on F^2*
Data / restraints / parameters	*8631 / 3 / 529*
Goodness-of-fit on F^2	*1.029*
Final R indices [I>2sigma(I)]	*R1 = 0.0513, wR2 = 0.1483*
R indices (all data)	*R1 = 0.0606, wR2 = 0.1590*
Absolute structure parameter	*0.5(3)*
Extinction coefficient	*n/a*
Largest diff. peak and hole	*0.452 and -0.413 e.A^-3*

SPECTRAL DATA:

^{1}H NMR of 1a:

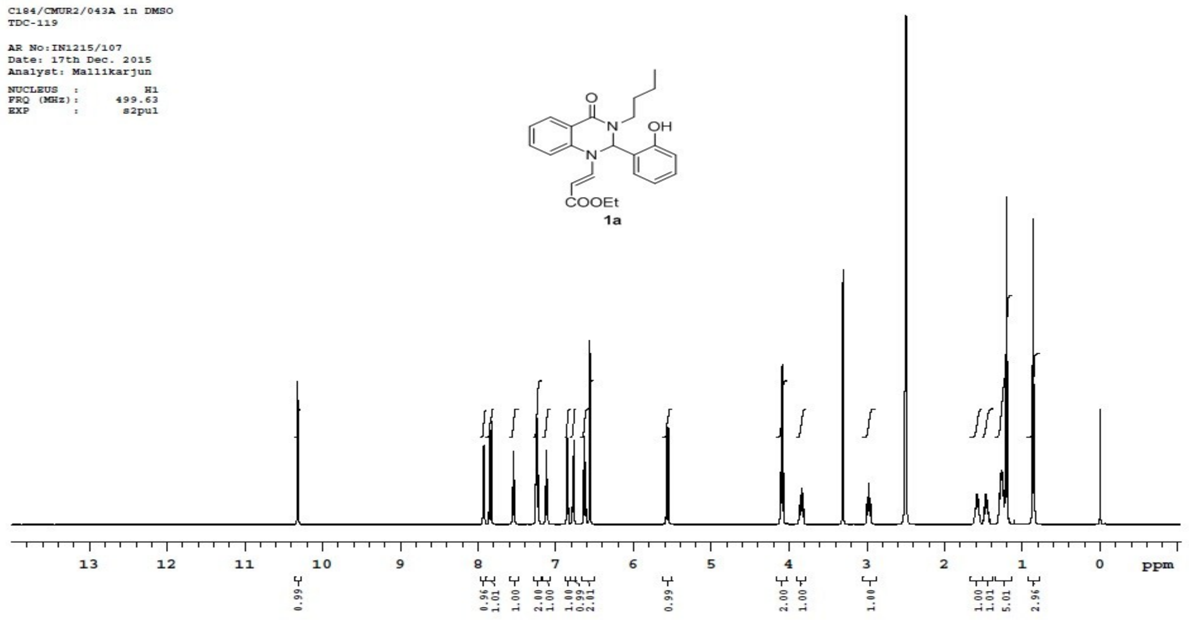

^{13}C NMR of 1a:

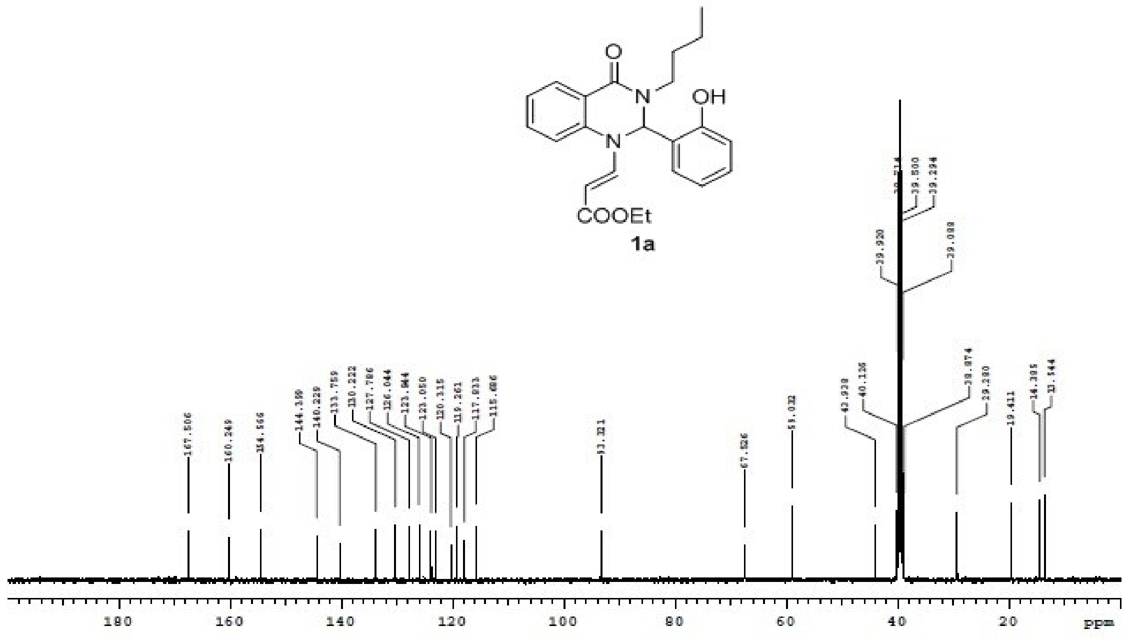

HRMS of 1a:

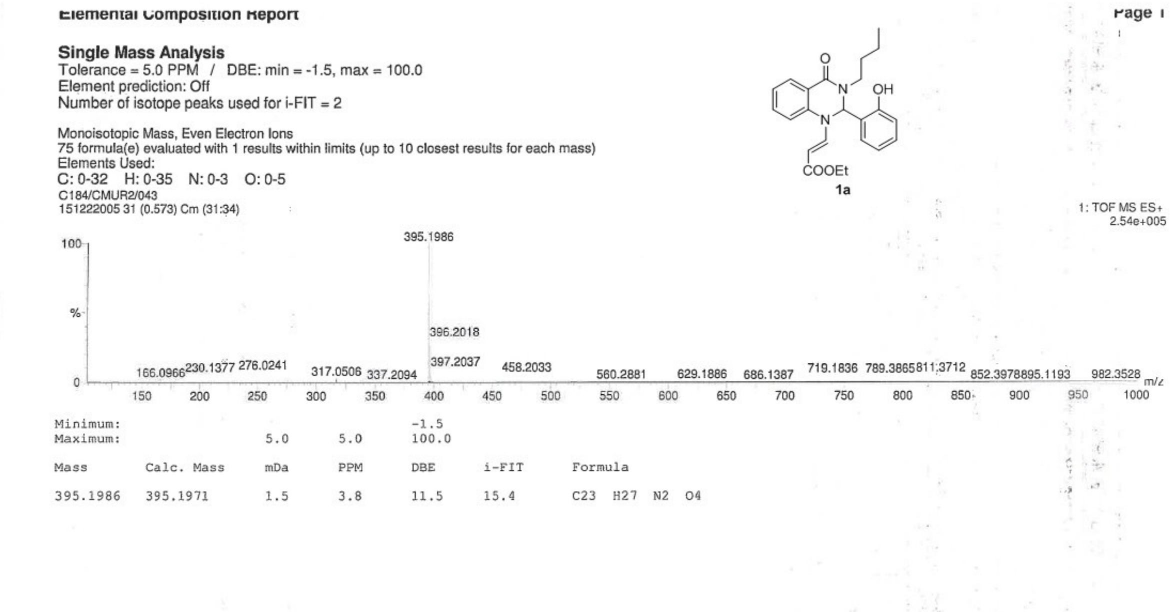
Elemental Composition Report

Page 1

Single Mass Analysis
Tolerance = 5.0 PPM / DBE: min = -1.5, max = 100.0
Element prediction: Off
Number of isotope peaks used for i-FIT = 2

Monoisotopic Mass, Even Electron Ions
75 formula(e) evaluated with 1 results within limits (up to 10 closest results for each mass)
Elements Used:
C: 0-32 H: 0-35 N: 0-3 O: 0-5
C184/CMUR2/043
151222005 31 (0.573) Cm (31:34)

Minimum:				-1.5		
Maximum:		5.0	5.0	100.0		
Mass	Calc. Mass	mDa	PPM	DBE	i-FIT	Formula
395.1986	395.1971	1.5	3.8	11.5	15.4	C23 H27 N2 O4

^{1}H NMR of 1b:

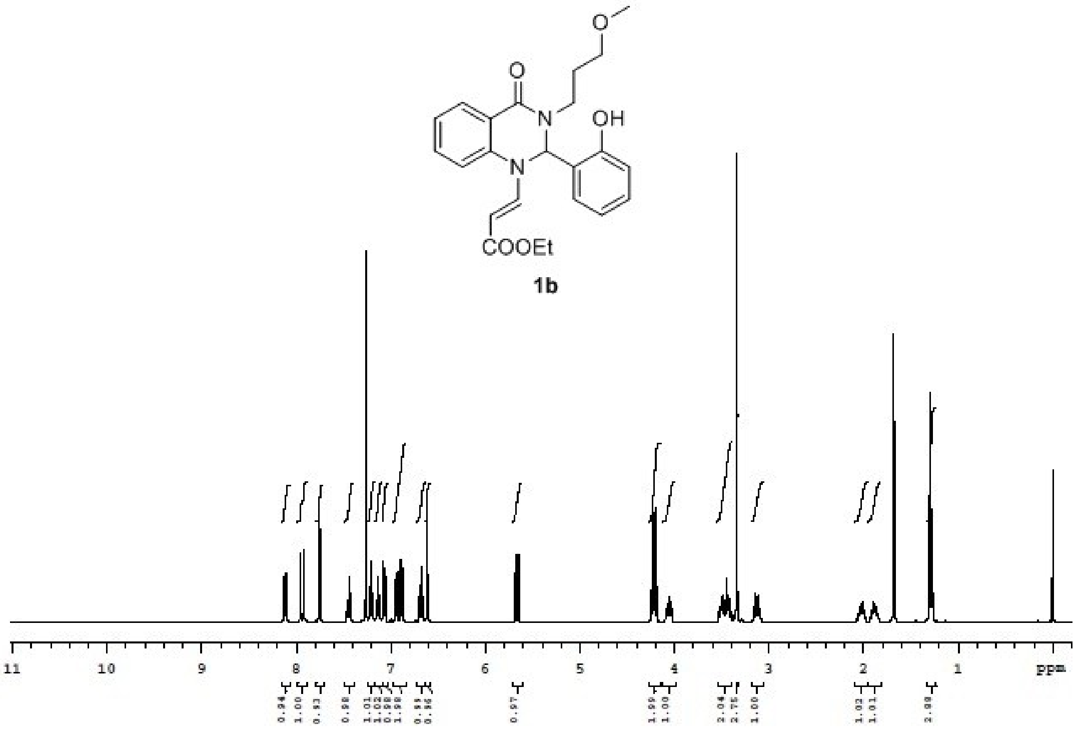

^{13}C NMR of 1b:

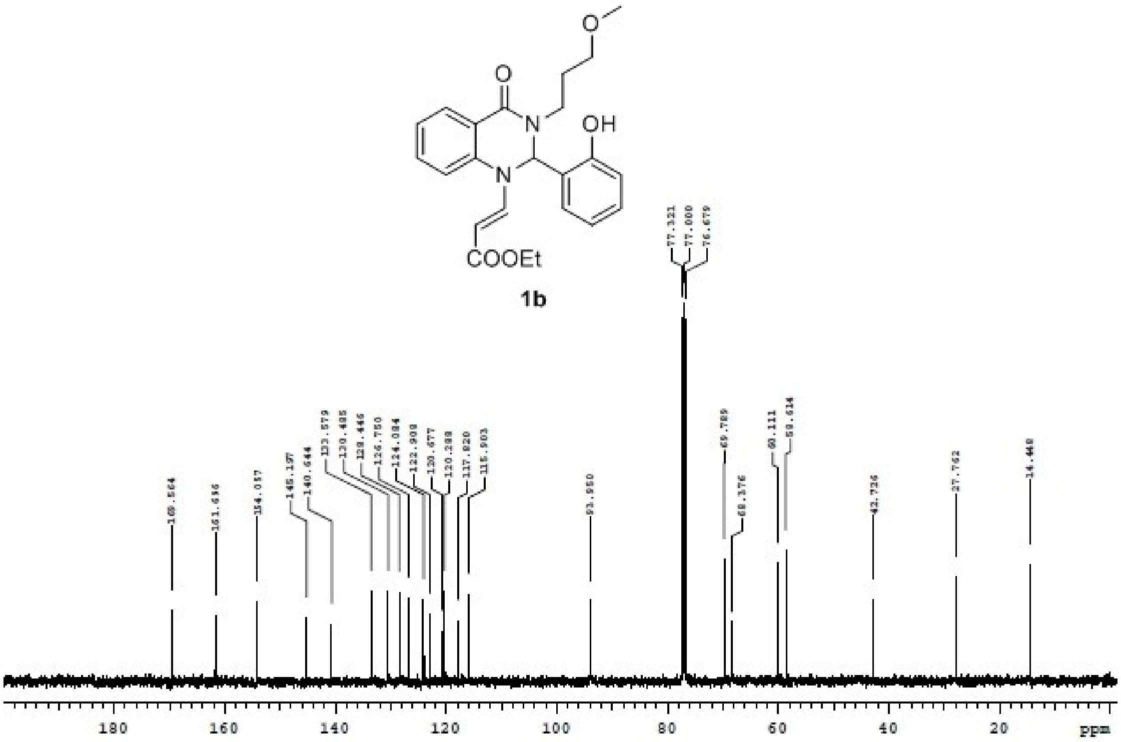

HRMS of 1b:

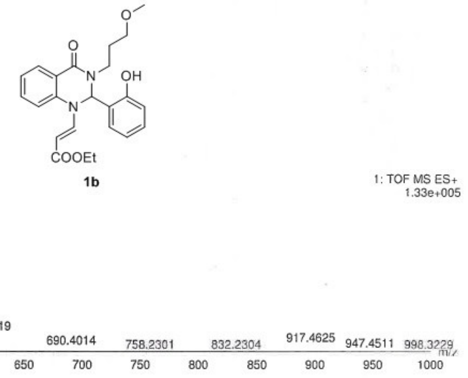

Elemental Composition Report

Page 1

Single Mass Analysis
Tolerance = 5.0 PPM / DBE: min = -1.5, max = 100.0
Element prediction: Off
Number of isotope peaks used for i-FIT = 2

Monoisotopic Mass, Even Electron Ions
50 formula(e) evaluated with 1 results within limits (up to 10 closest results for each mass)
Elements Used:
C: 0-32 H: 0-35 N: 0-3 O: 0-5
C446/CSIL2/022
151222004 25 (0.473) Cm (25:29)

Minimum:				-1.5		
Maximum:		5.0	5.0	100.0		

Mass	Calc. Mass	mDa	PPM	DBE	i-FIT	Formula
395.1601	395.1603	-0.2	-0.6	10.5	3.9	C23 H27 N2 O4

^{1}H NMR of 1c:

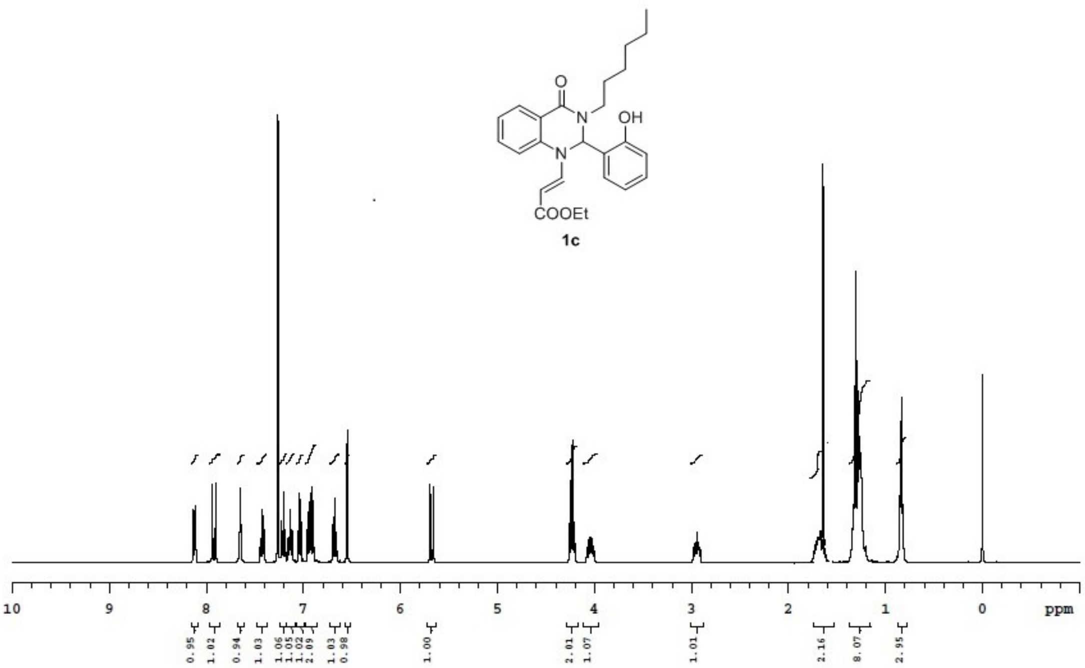

^{13}C NMR of 1c:

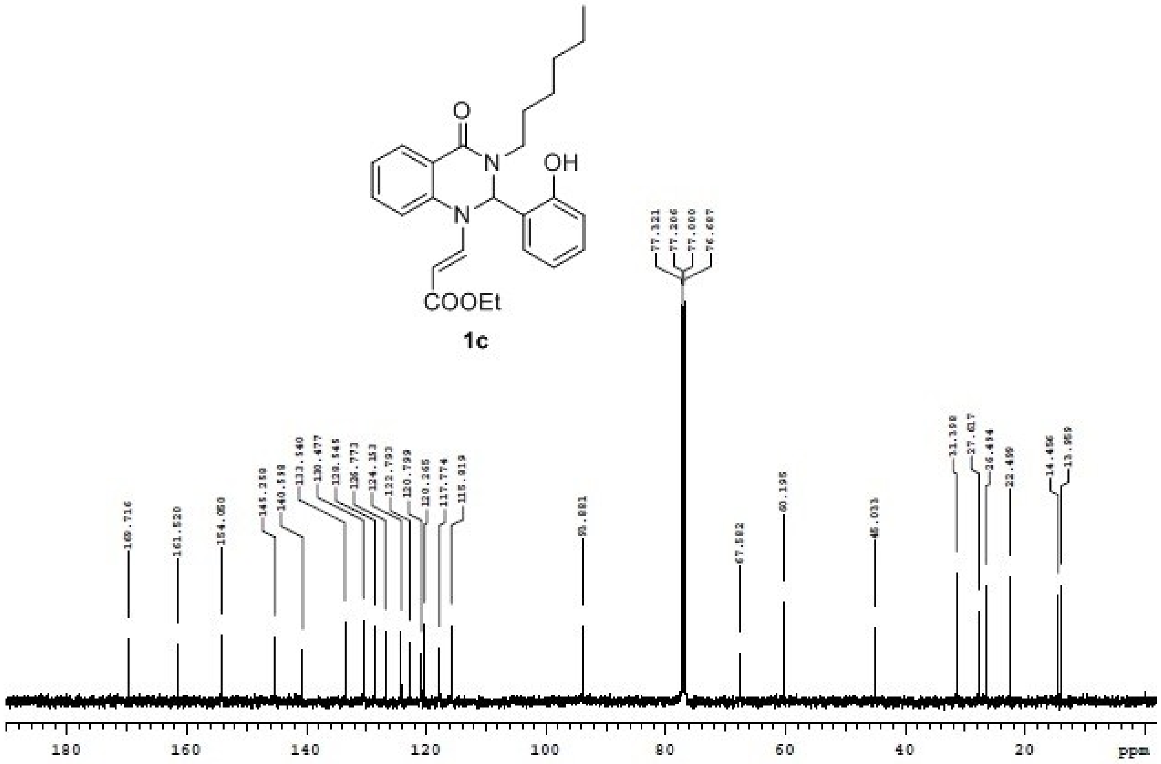

HRMS of 1c:

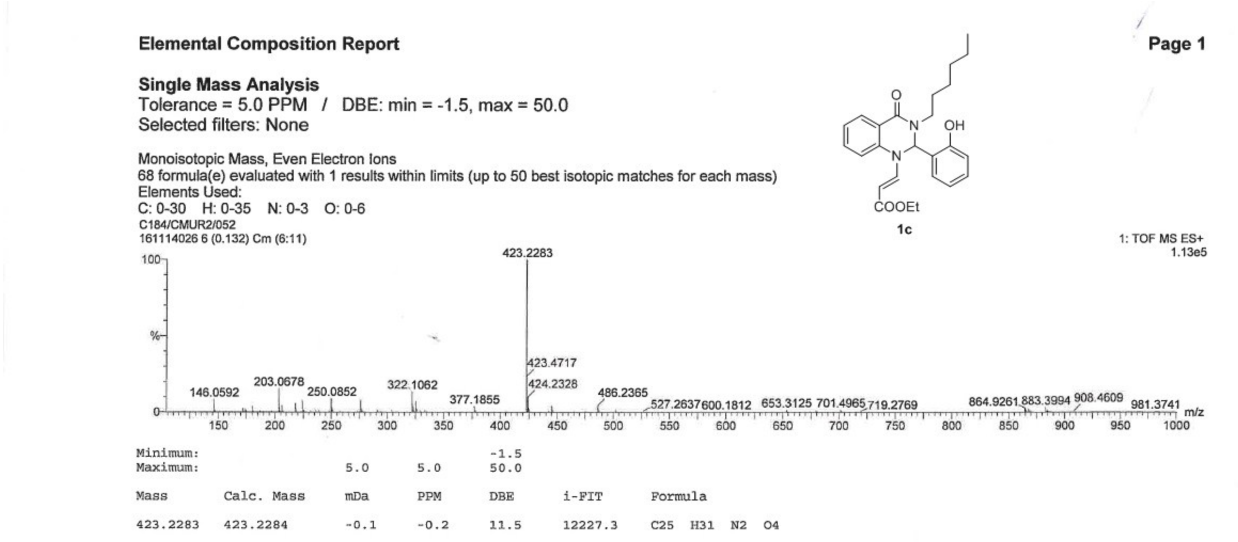

^{1}H NMR of 1d:

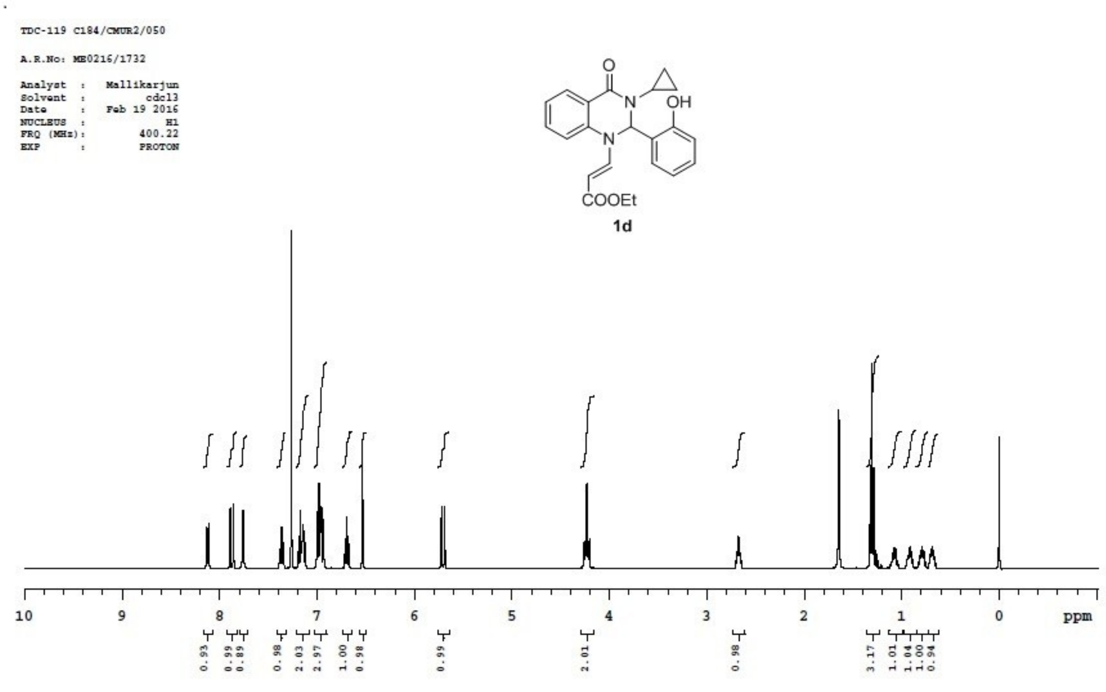

^{13}C NMR of 1d:

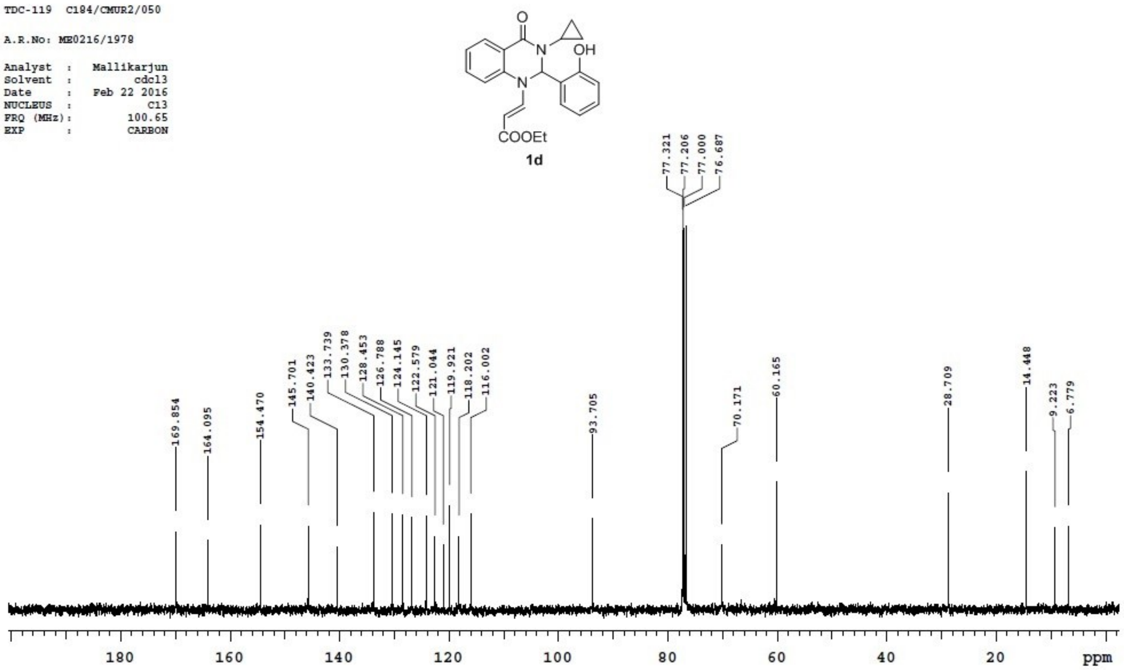

HRMS of 1d:

Elemental Composition Report Page 1

Single Mass Analysis
Tolerance = 5.0 PPM / DBE: min = -1.5, max = 50.0
Selected filters: None

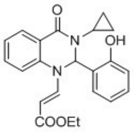

1d

Monoisotopic Mass, Even Electron Ions
87 formula(e) evaluated with 1 results within limits (up to 50 best isotopic matches for each mass)
Elements Used:
C: 0-30 H: 0-35 N: 0-3 O: 0-6
C184/CMUR2/050
161114023 5 (0.118) Cm (3:10-33:38)

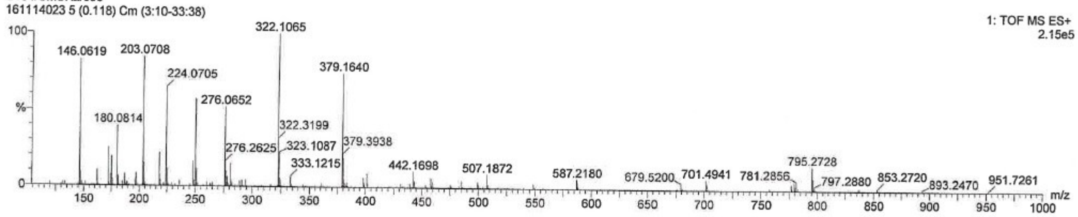

Minimum:				-1.5		
Maximum:		5.0	5.0	50.0		
Mass	Calc. Mass	mDa	PPM	DBE	i-FIT	Formula
379.1640	379.1658	-1.8	-4.7	12.5	15.9	C22 H23 N2 O4

^{1}H NMR of 1e:

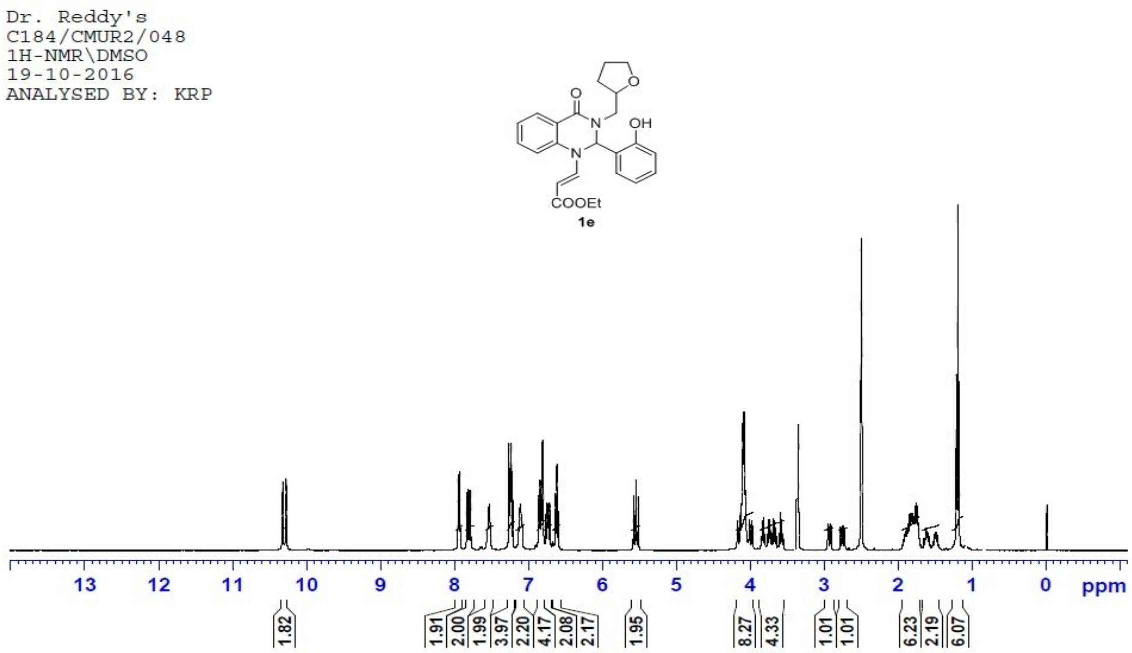

^{13}C NMR of 1e:

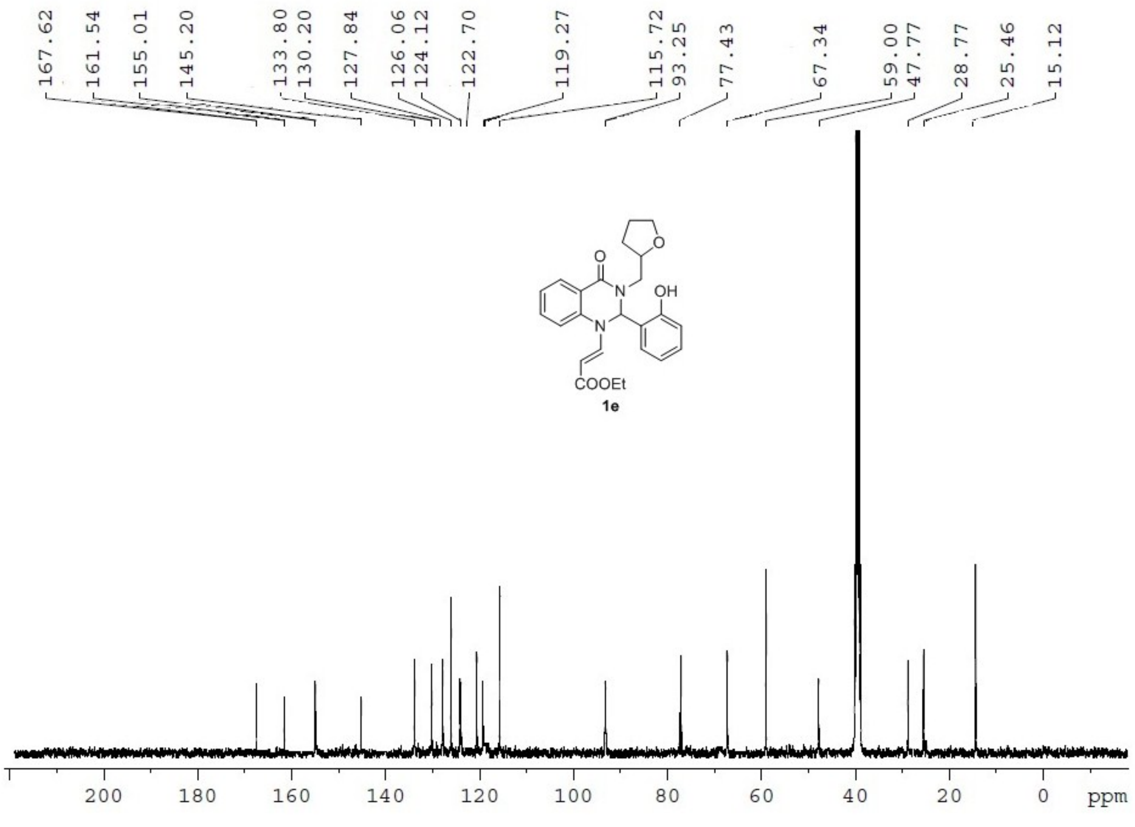

HRMS of 1e:

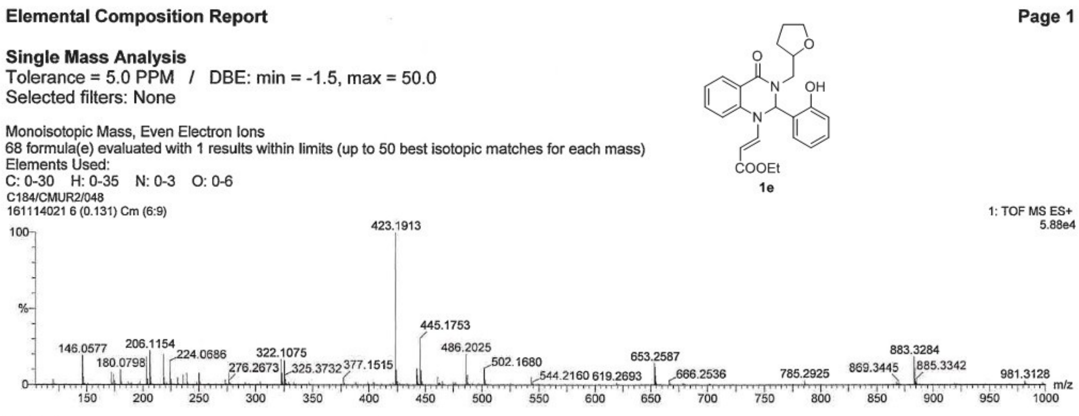

Minimum:				-1.5		
Maximum:		5.0	5.0	50.0		
Mass	Calc. Mass	mDa	PPM	DBE	i-FIT	Formula
423.1913	423.1920	-0.7	-1.7	12.5	5768.2	C24 H27 N2 O5

^{1}H NMR of 1f:

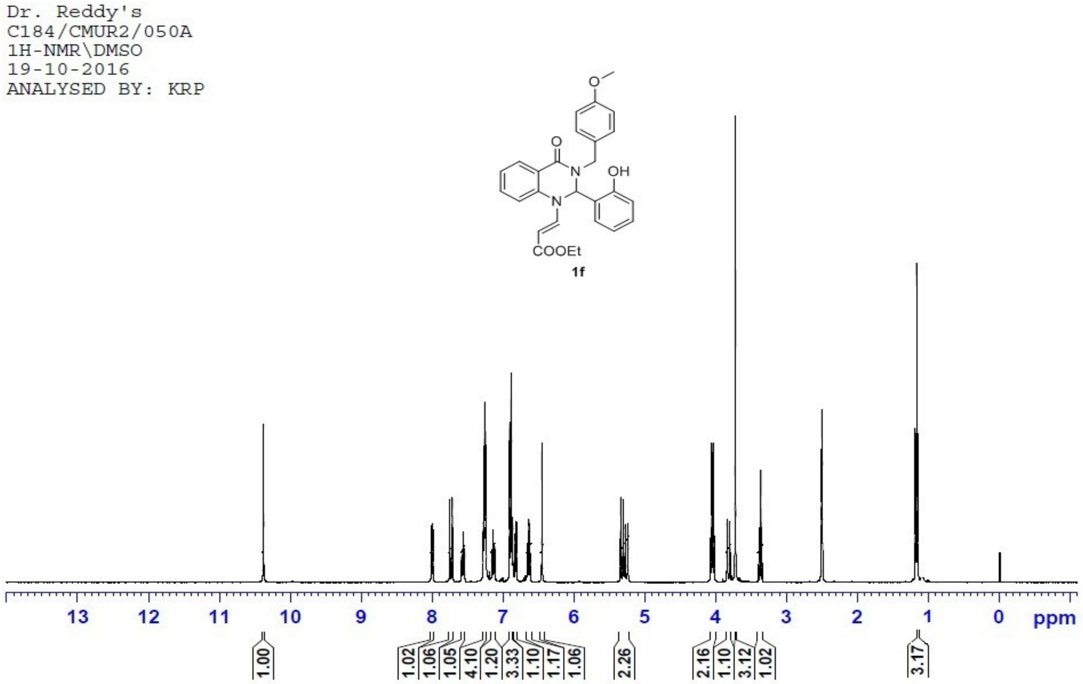

^{13}C NMR of 1f:

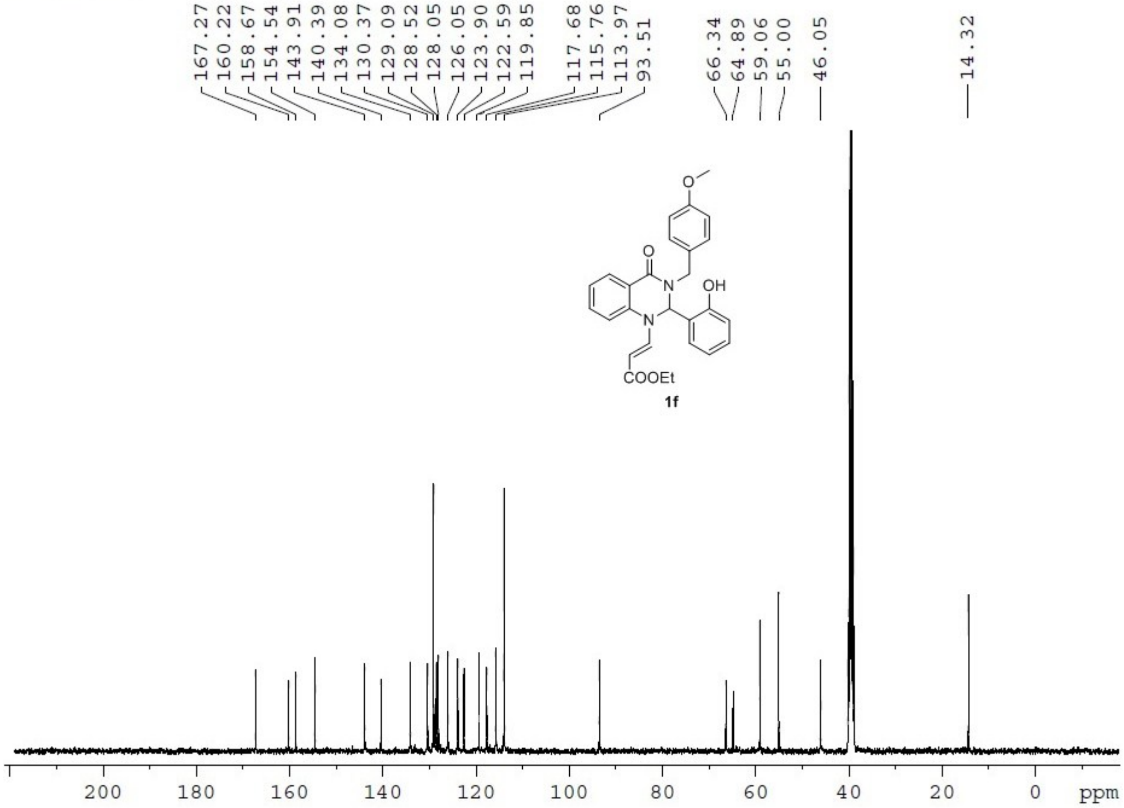

HRMS of 1f:

Elemental Composition Report **Page 1**

Single Mass Analysis
Tolerance = 5.1 PPM / DBE: min = -1.5, max = 50.0
Selected filters: None

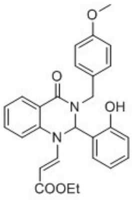

Monoisotopic Mass, Even Electron Ions
42 formula(e) evaluated with 1 results within limits (up to 50 best isotopic matches for each mass)
Elements Used:
C: 0-30 H: 0-35 N: 0-3 O: 0-6
C184/CMUR2/050 A
161114024 11 (0.251) Cm (11:12)

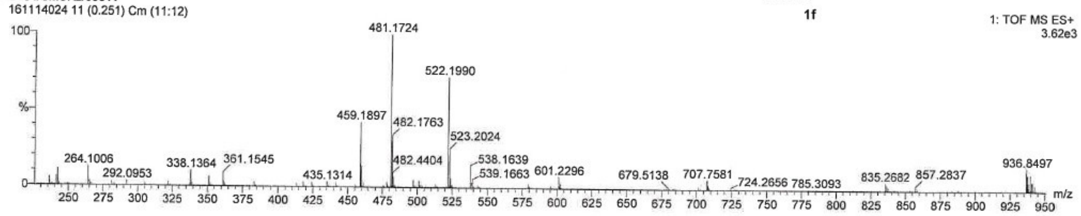

Minimum:				-1.5		
Maximum:		5.0	5.1	50.0		
Mass	Calc. Mass	mDa	PPM	DBE	i-FIT	Formula
459.1897	459.1920	-2.3	-5.0	15.5	1.7	C27 H27 N2 O5

^{1}H NMR of 1g:

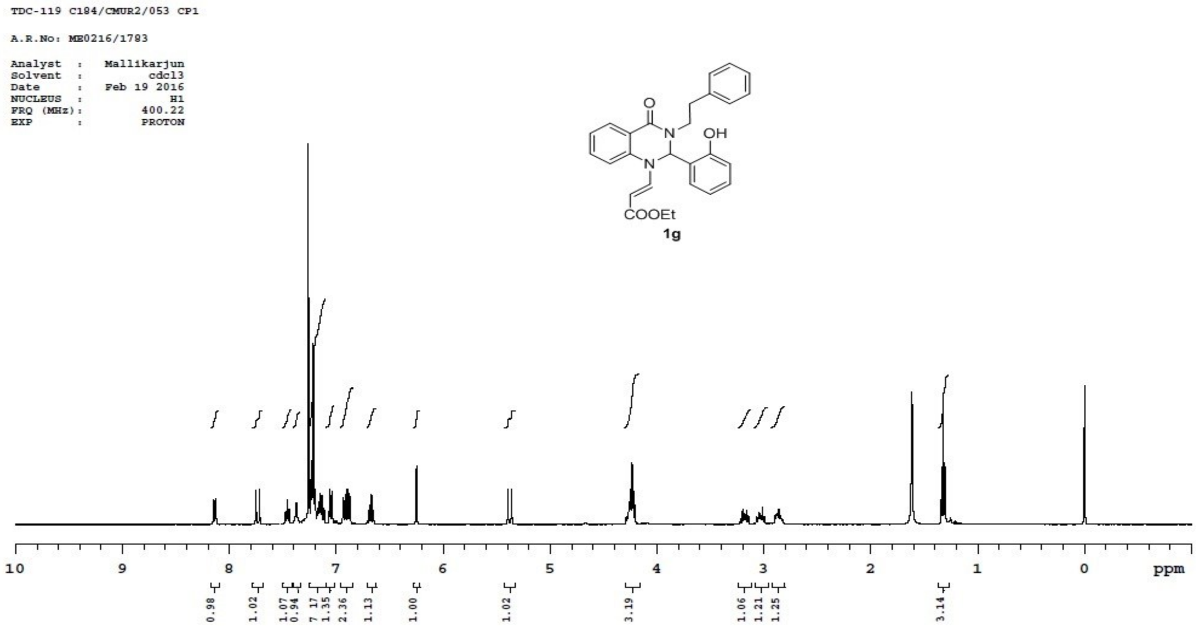

^{13}C NMR of 1g:

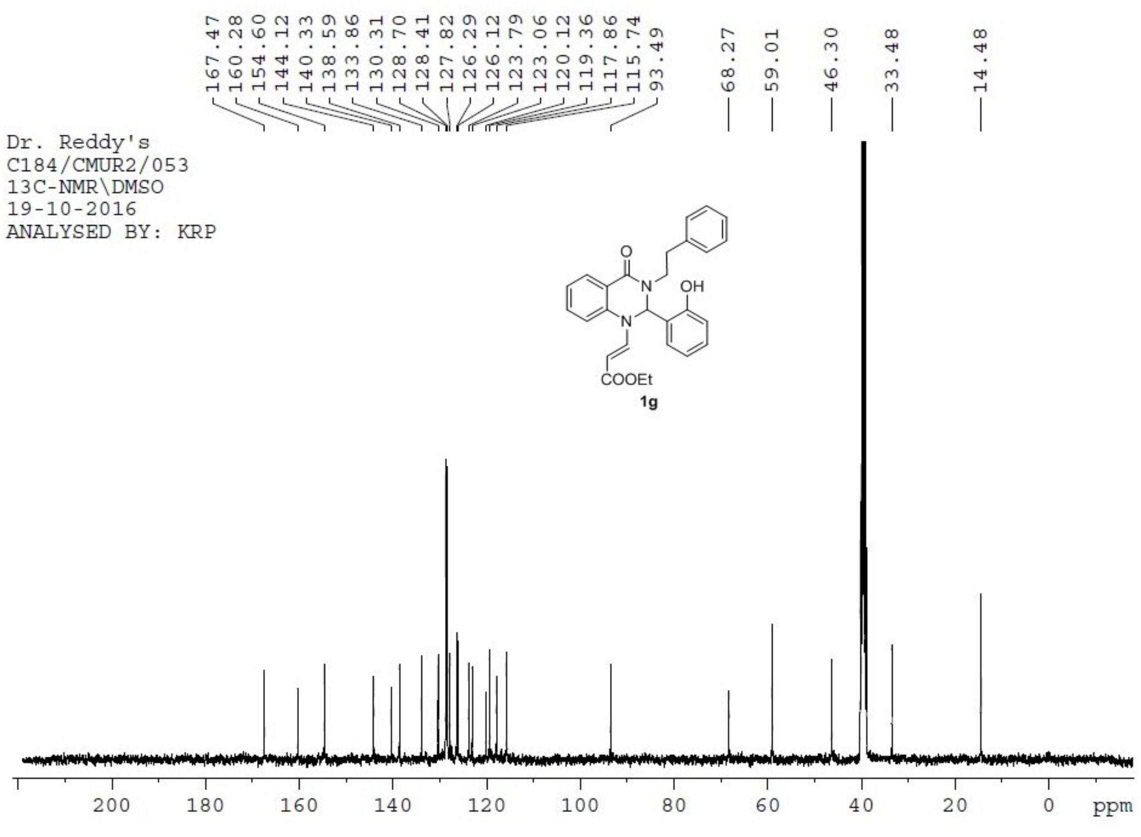

HRMS of 1g:

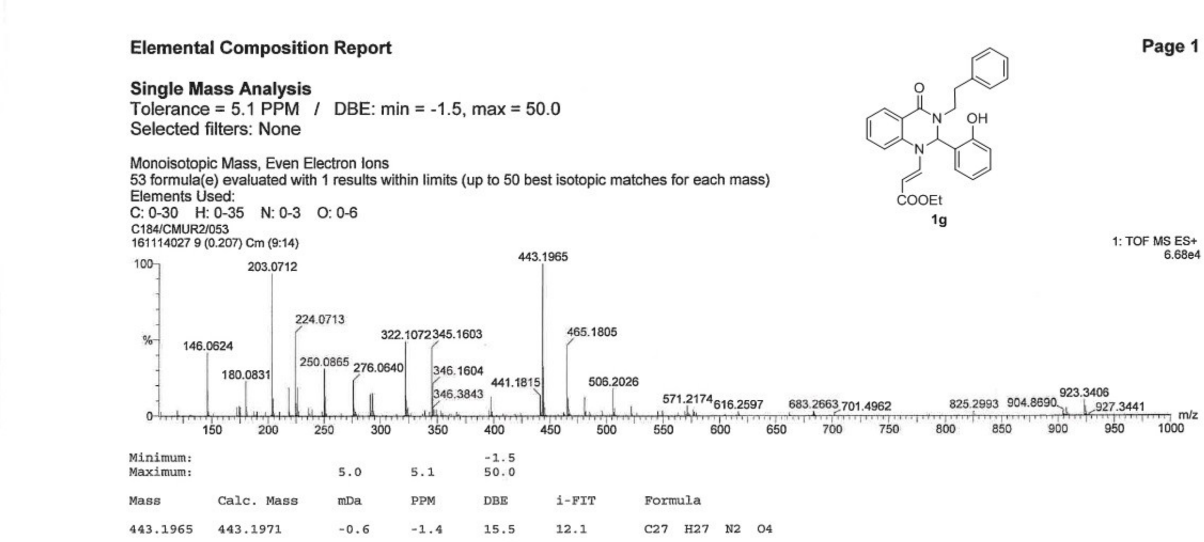

^{1}H NMR of 1h:

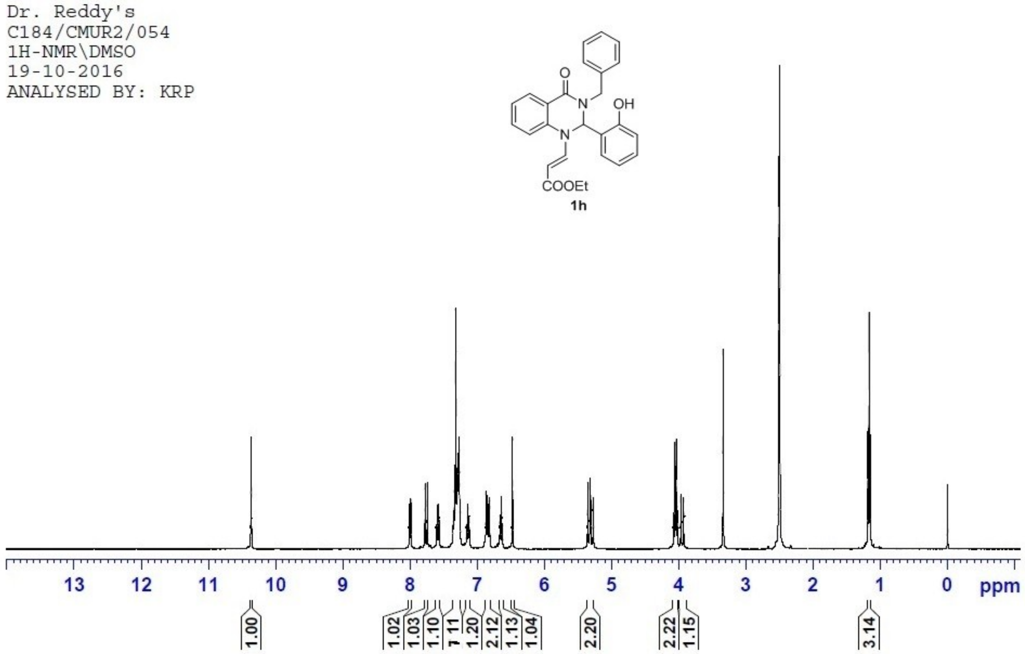

^{13}C NMR of 1h:

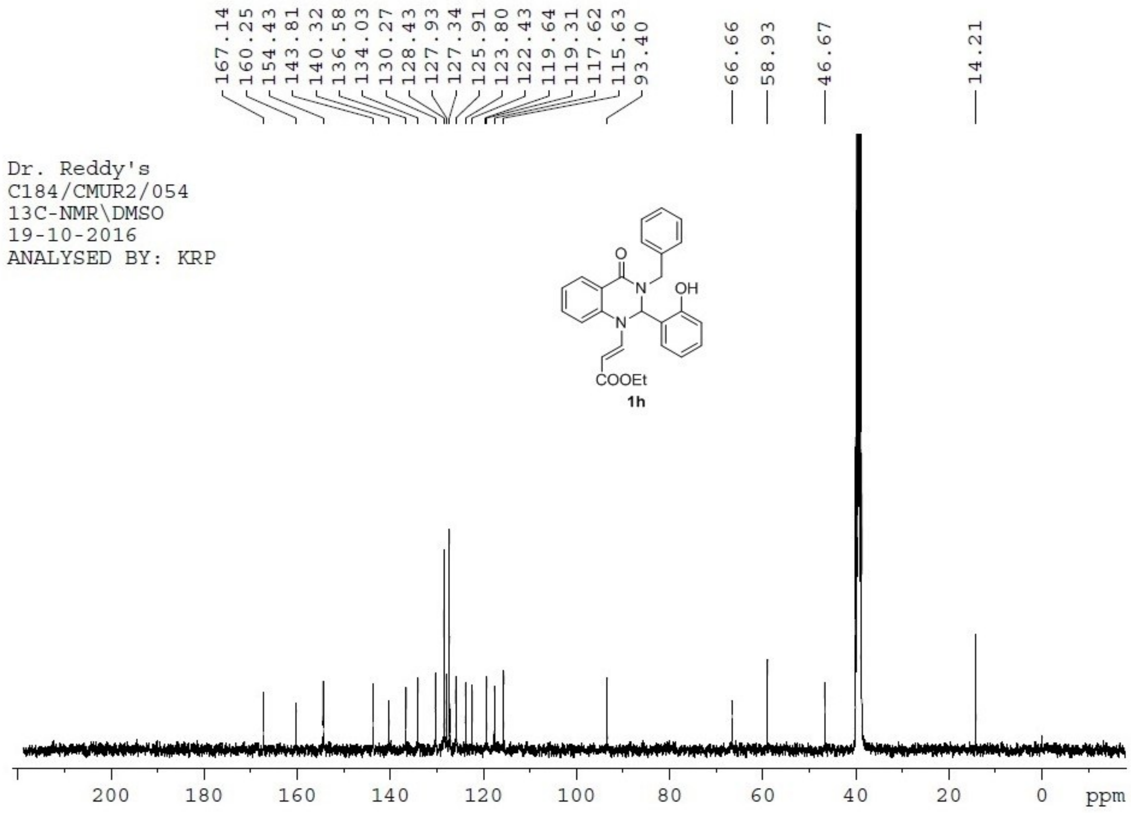

HRMS of 1h:

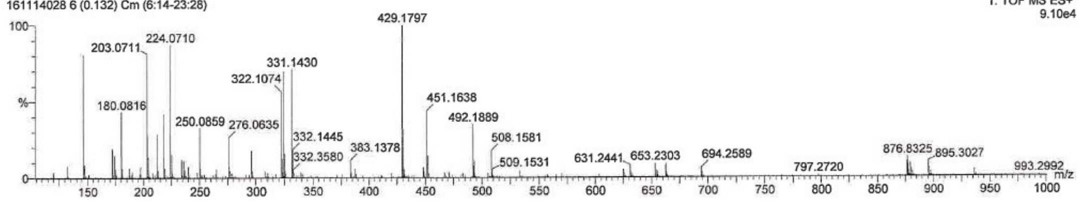

Elemental Composition Report Page 1

Single Mass Analysis
Tolerance = 5.0 PPM / DBE: min = -1.5, max = 50.0
Selected filters: None

Monoisotopic Mass, Even Electron Ions
62 formula(e) evaluated with 1 results within limits (up to 50 best isotopic matches for each mass)
Elements Used:
C: 0-30 H: 0-35 N: 0-3 O: 0-6
C184/CMUR2/054
161114028 6 (0.132) Cm (6:14-23:28)

Minimum:				-1.5		
Maximum:		5.0	5.0	50.0		
Mass	Calc. Mass	mDa	PPM	DBE	i-FIT	Formula
429.1797	429.1814	-1.7	-4.0	15.5	39.2	C26 H25 N2 O4

^{1}H NMR of 1i:

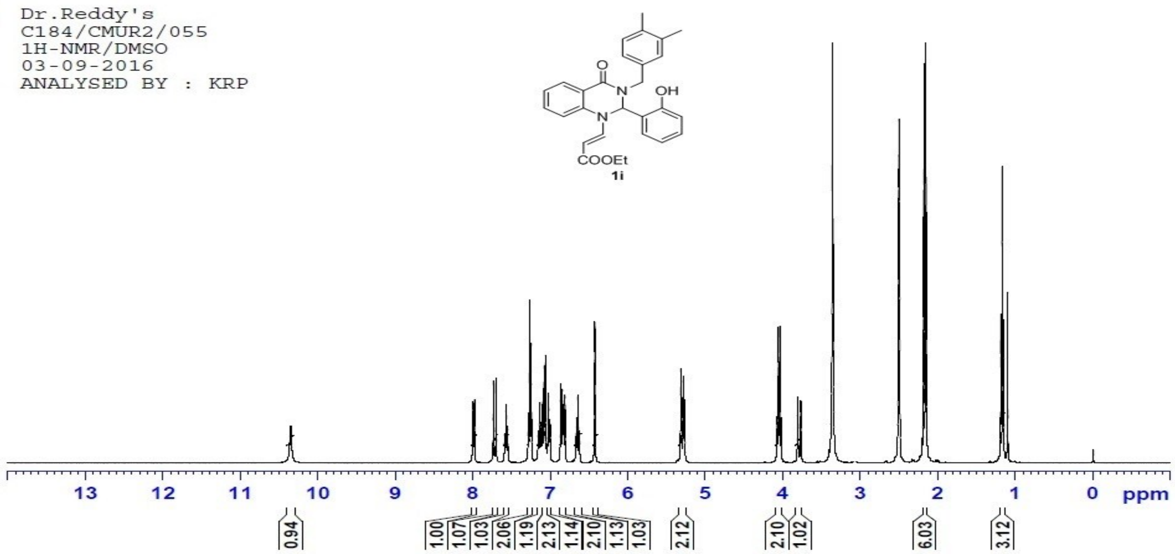

^{13}C NMR of 1i:

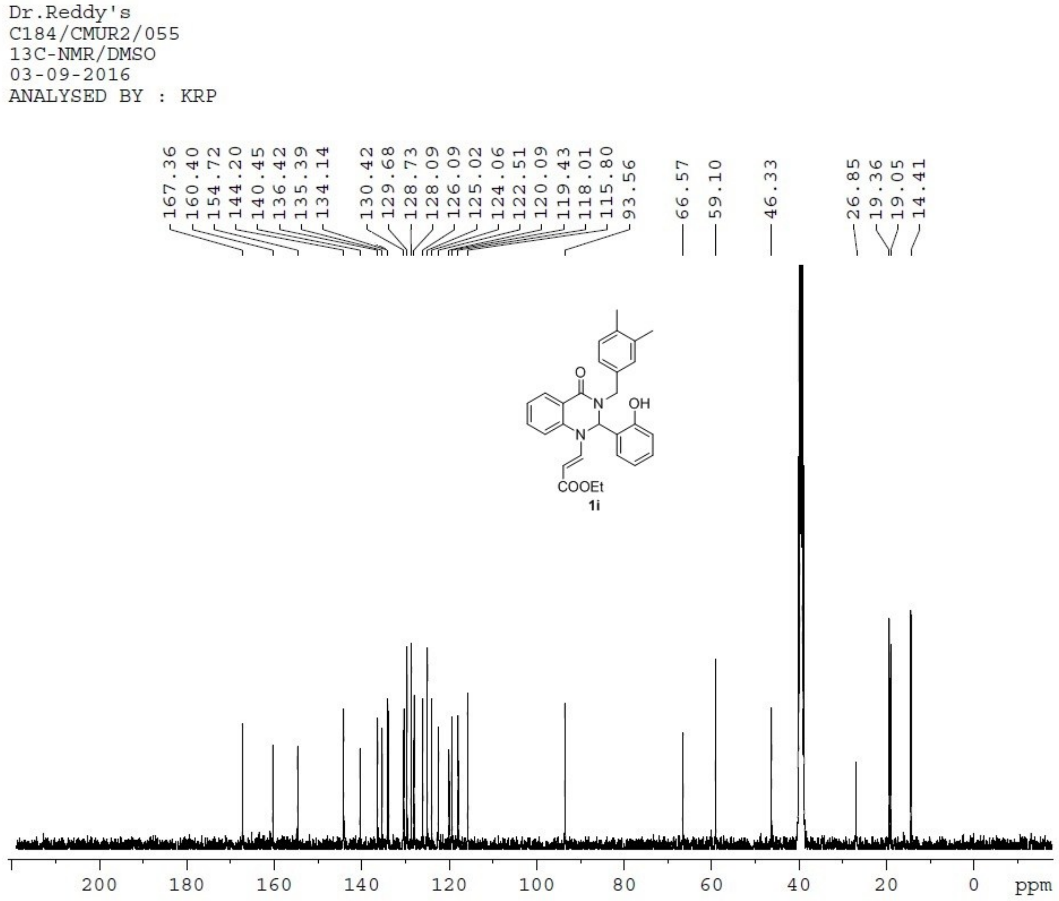

HRMS of 1i:

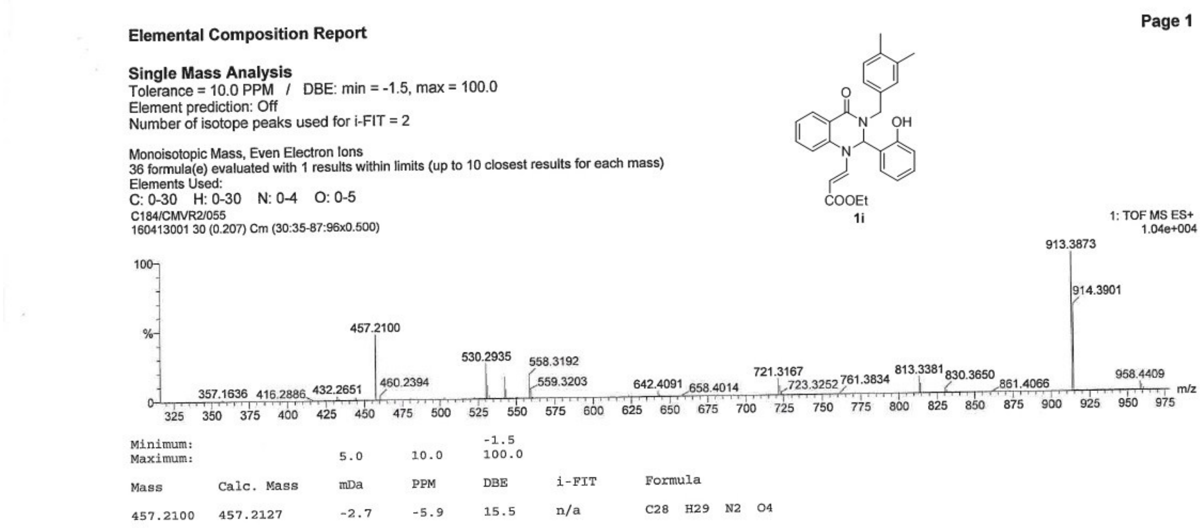

^{1}H NMR of 1j:

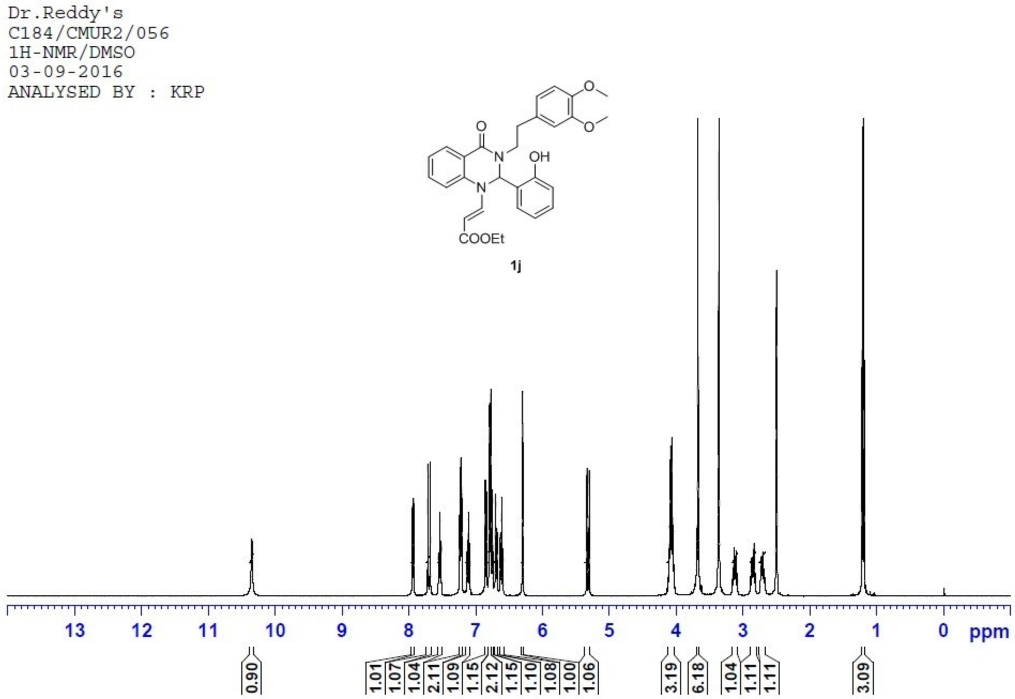

^{13}C NMR of 1j:

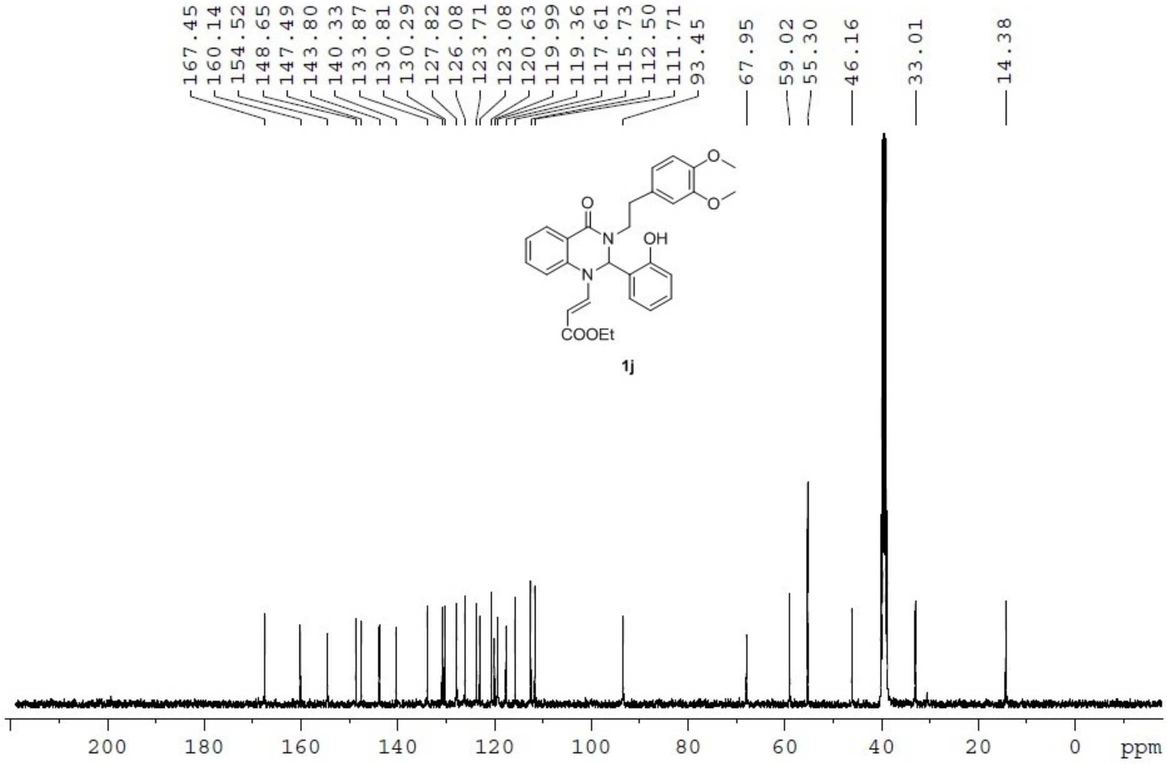

HRMS of 1j:

Elemental Composition Report **Page 1**

Single Mass Analysis
Tolerance = 5.0 PPM / DBE: min = -1.5, max = 50.0
Selected filters: None

Monoisotopic Mass, Even Electron Ions
23 formula(e) evaluated with 1 results within limits (up to 50 best isotopic matches for each mass)
Elements Used:
C: 0-30 H: 0-35 N: 0-3 O: 0-6
C184/CMUR2/056
161114029 5 (0.119) Cm (5:11-45:46)

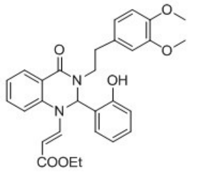

1j

1: TOF MS ES+
2.92e5

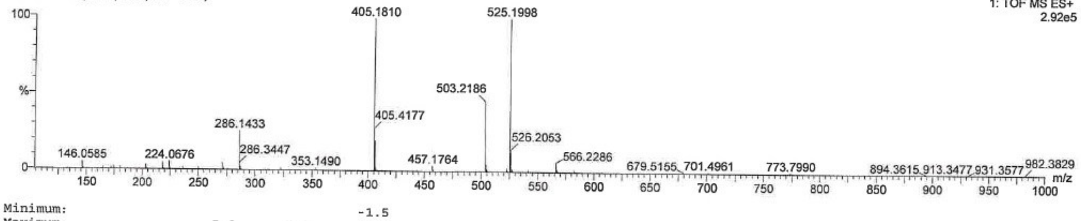

Minimum:				-1.5		
Maximum:		5.0	5.0	50.0		
Mass	Calc. Mass	mDa	PPM	DBE	i-FIT	Formula
503.2186	503.2182	0.4	0.8	15.5	16811.2	C29 H31 N2 O6

^{1}H NMR of 1k:

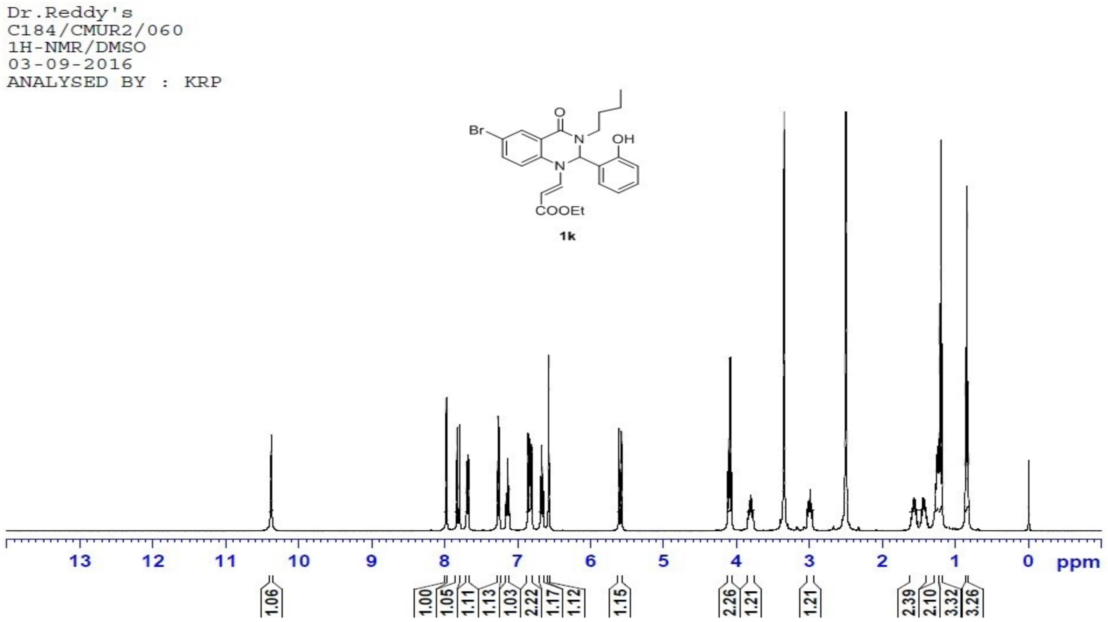

^{13}C NMR of 1k:

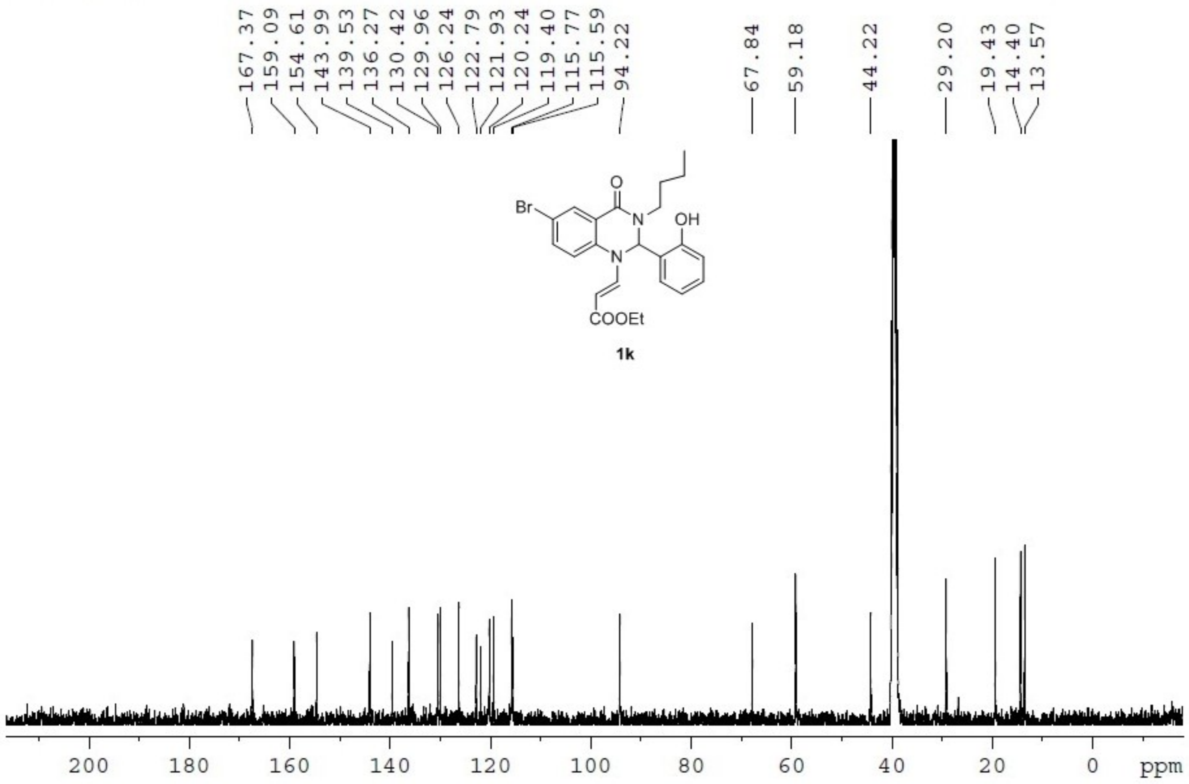

HRMS of 1k:

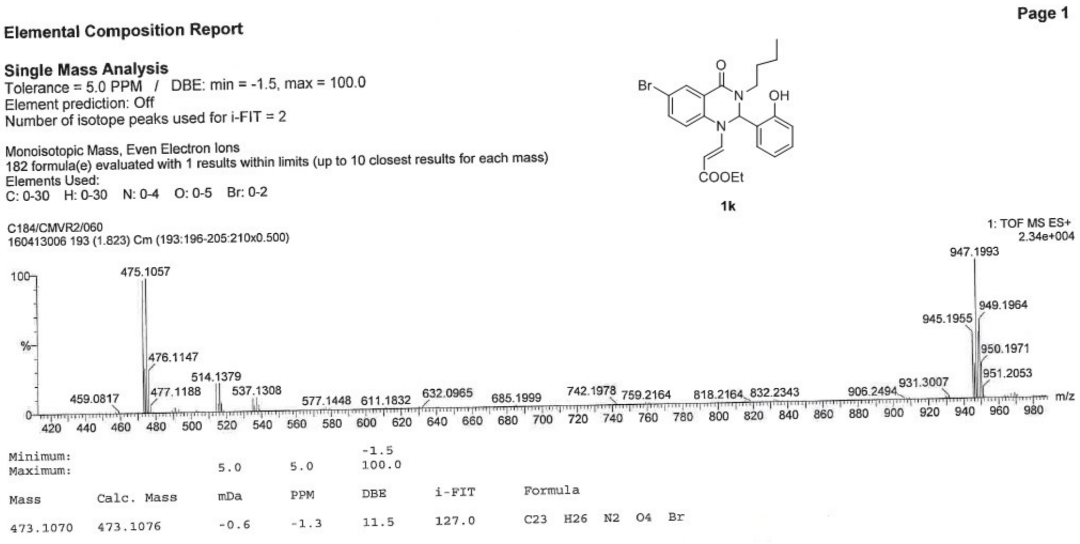

^{1}H NMR of 1l:

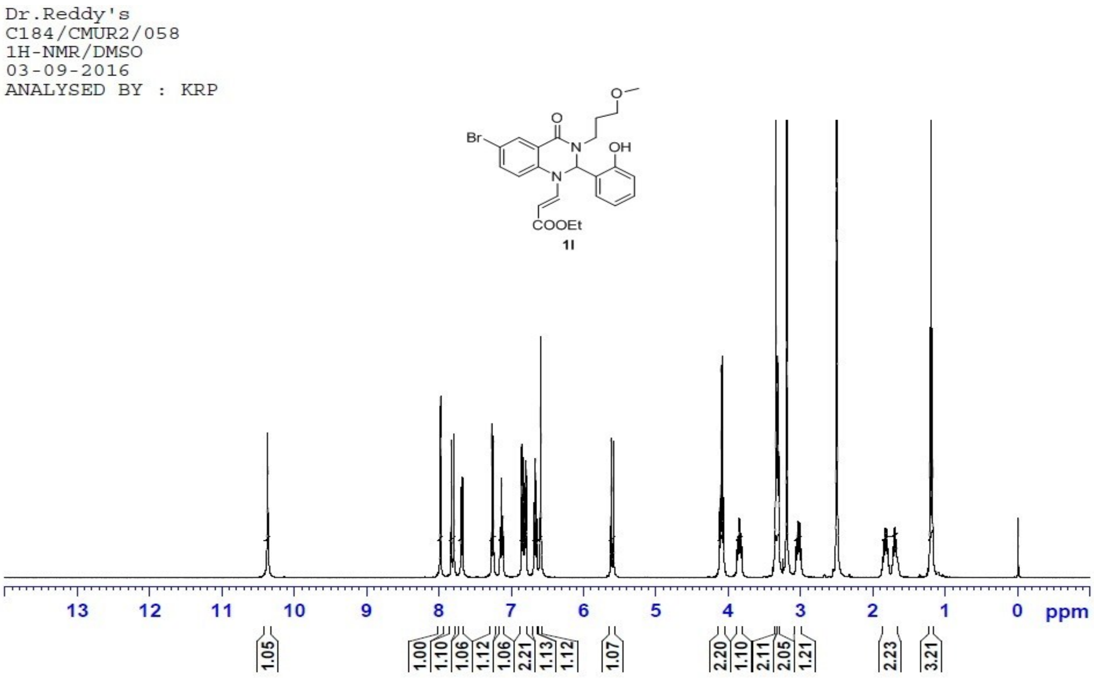

^{13}C NMR of 1l:

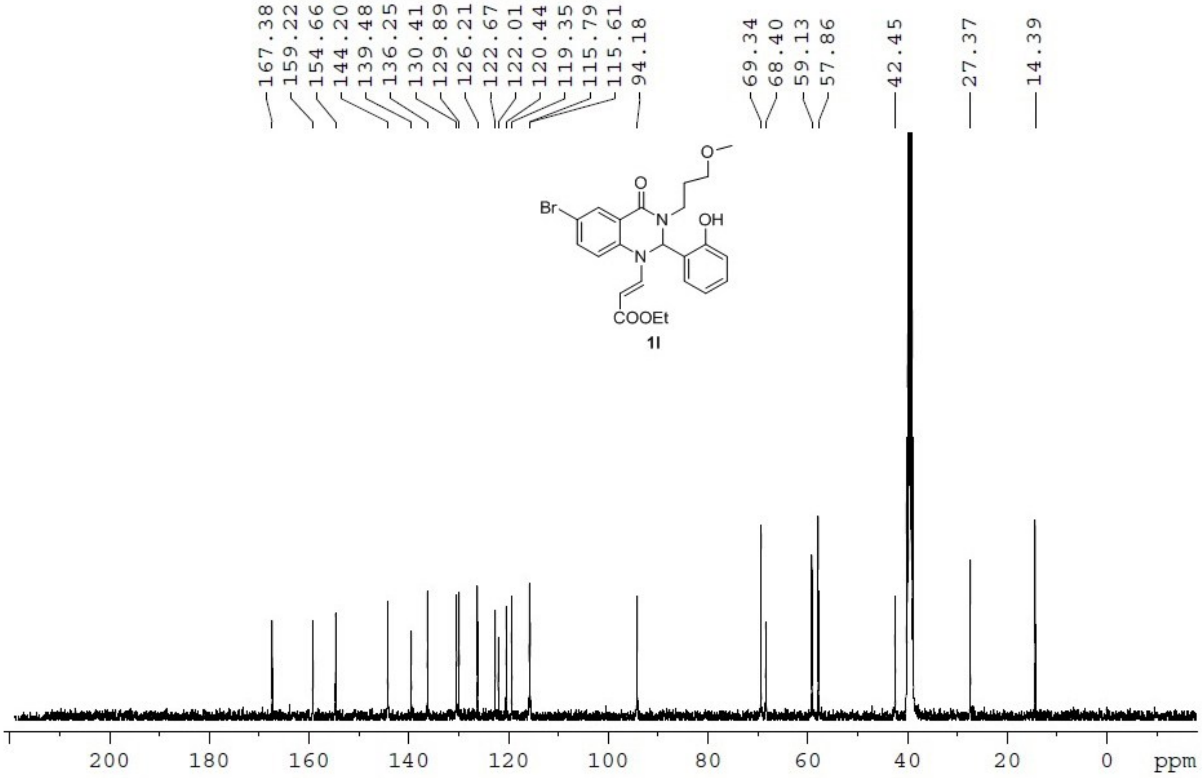

HRMS of 1l:

Elemental Composition Report Page 1

Single Mass Analysis
Tolerance = 5.0 PPM / DBE: min = -1.5, max = 100.0
Element prediction: Off
Number of isotope peaks used for i-FIT = 2

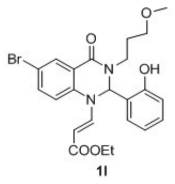

Monoisotopic Mass, Even Electron Ions
130 formula(e) evaluated with 1 results within limits (up to 10 closest results for each mass)
Elements Used:
C: 0-25 H: 0-27 N: 0-4 O: 0-5 Br: 0-2

C184/CMVR2/058
160413004 174 (1.641) Cm (169:174-194:200x0.500)

1: TOF MS ES+
1.78e+004

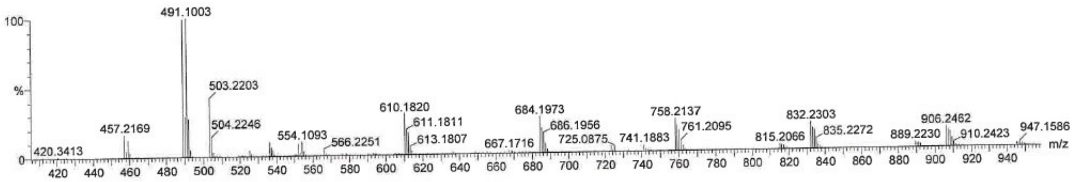

		mDa	PPM	DBE	i-FIT	Formula
Minimum:				-1.5		
Maximum:		5.0	5.0	100.0		
Mass	Calc. Mass	mDa	PPM	DBE	i-FIT	Formula
489.1013	489.1025	-1.2	-2.5	11.5	16.2	C23 H26 N2 O5 Br

^{1}H NMR of 1m:

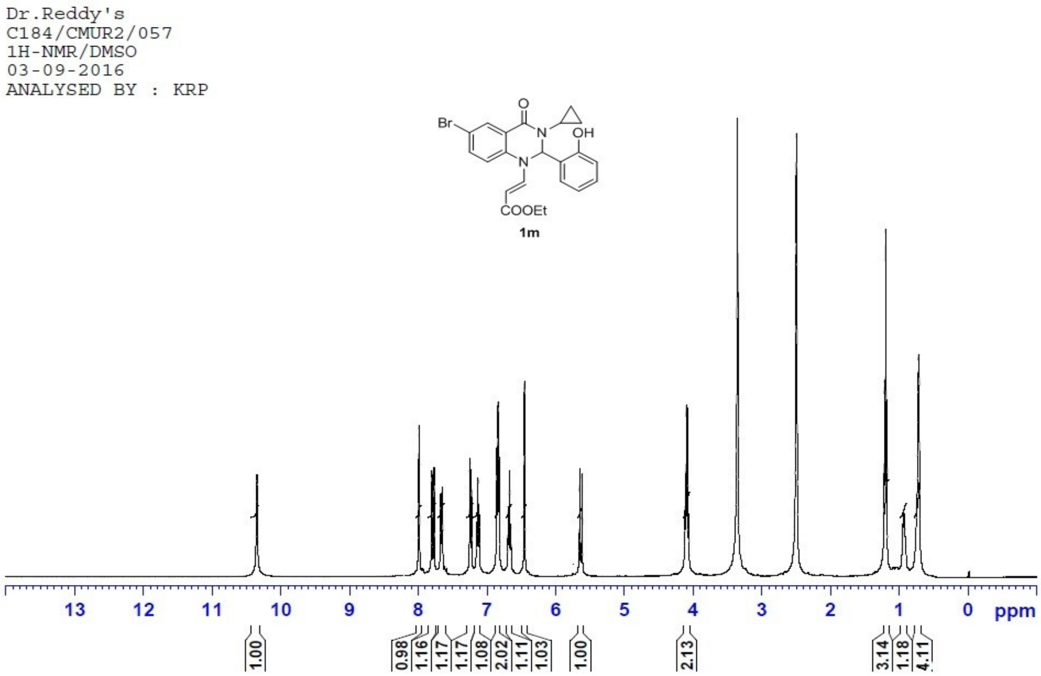

^{13}C NMR of 1m:

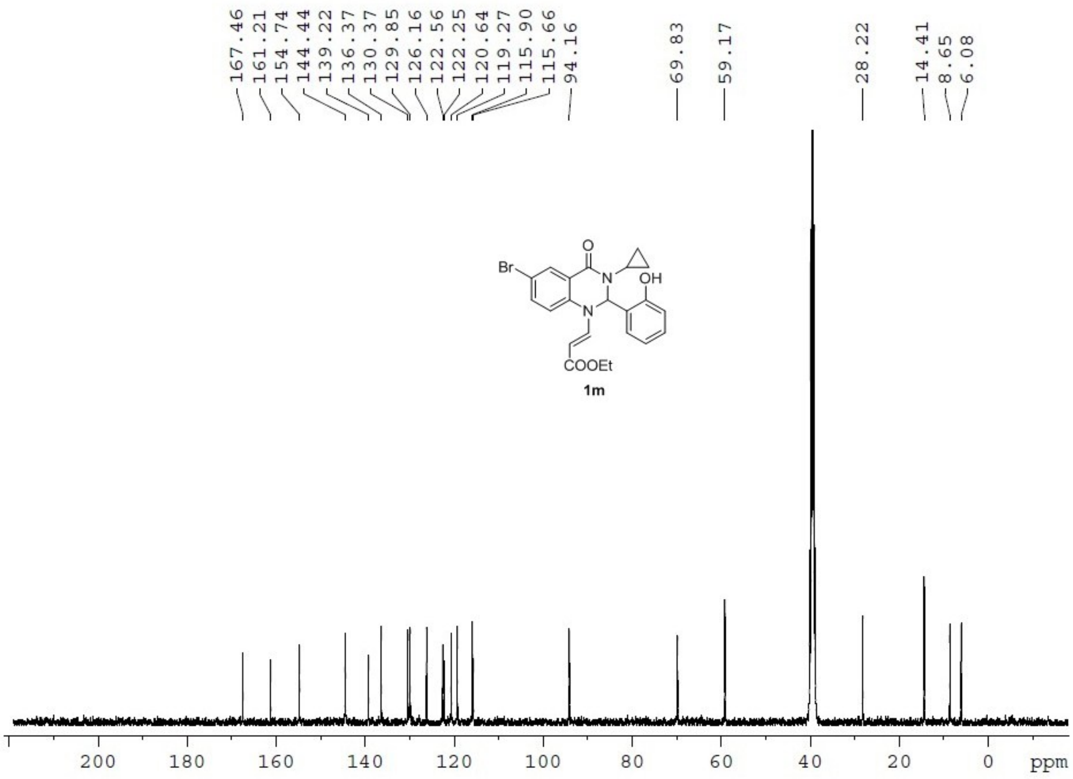

ESMS of 1m:

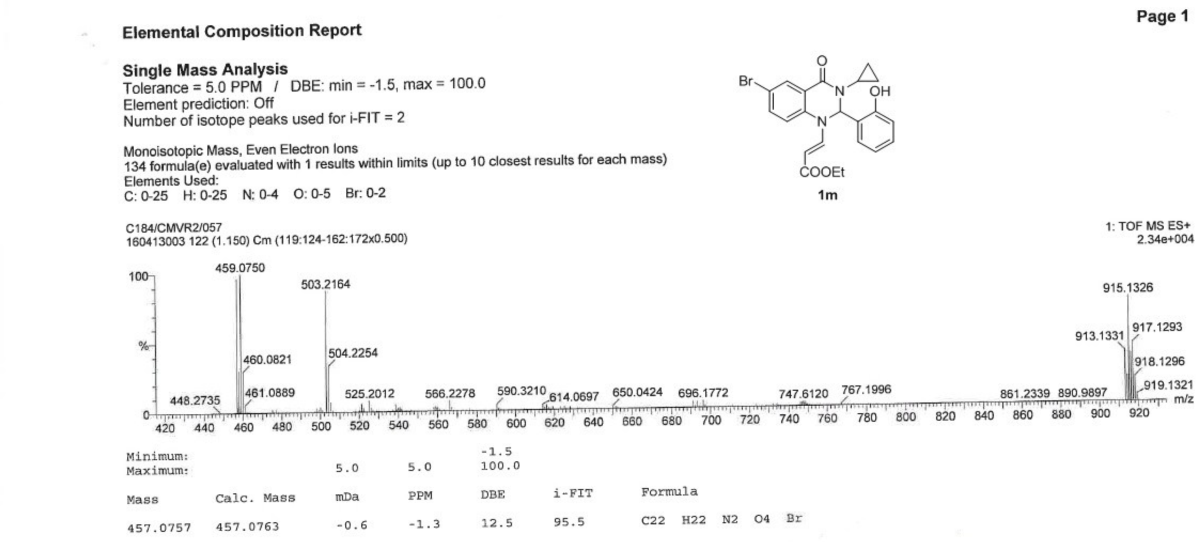

¹H NMR of 1n:

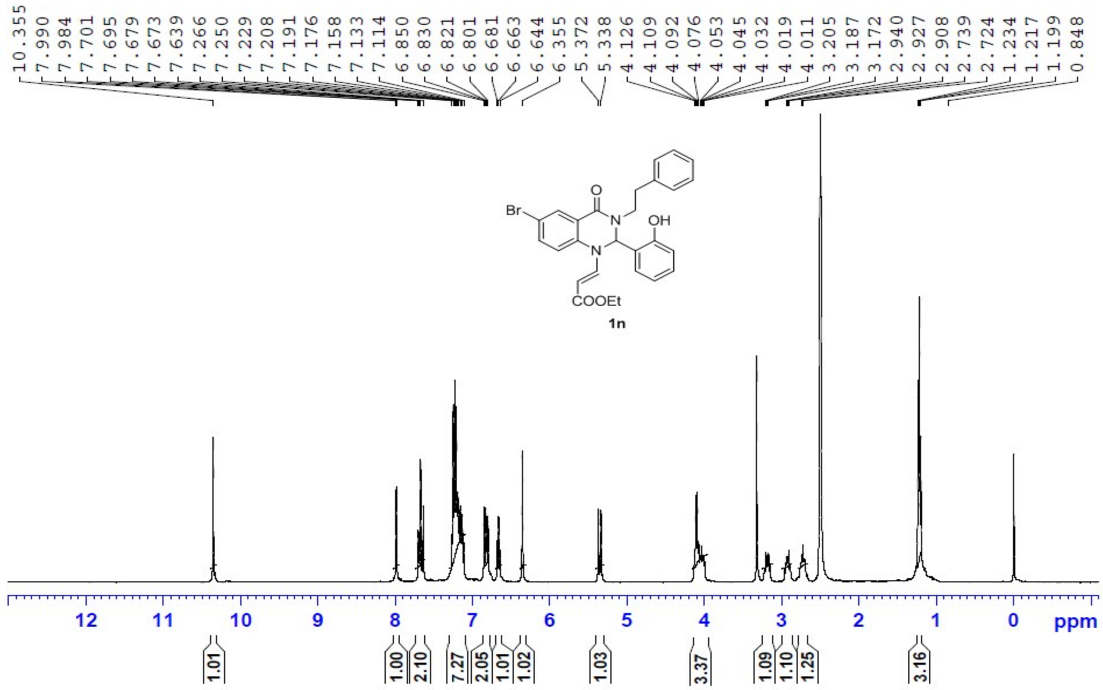

^{13}C NMR of 1n:

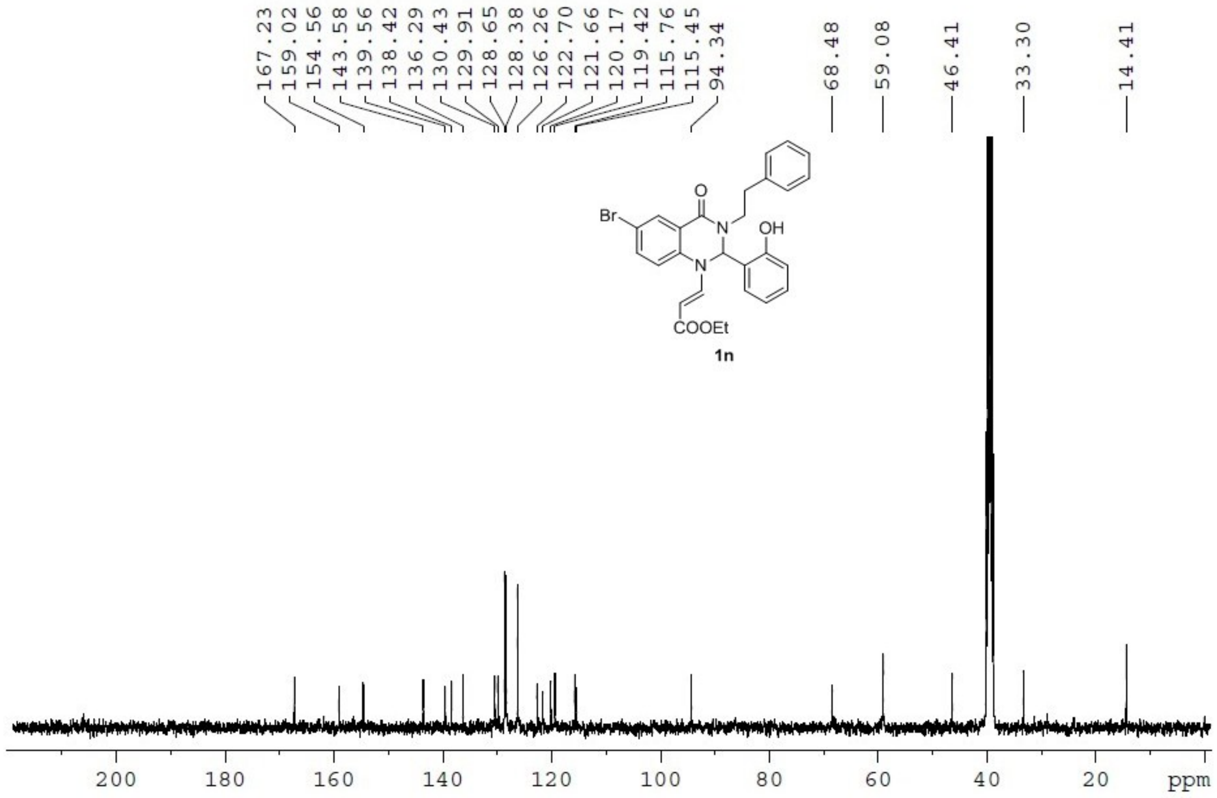

ESMS of 1n:

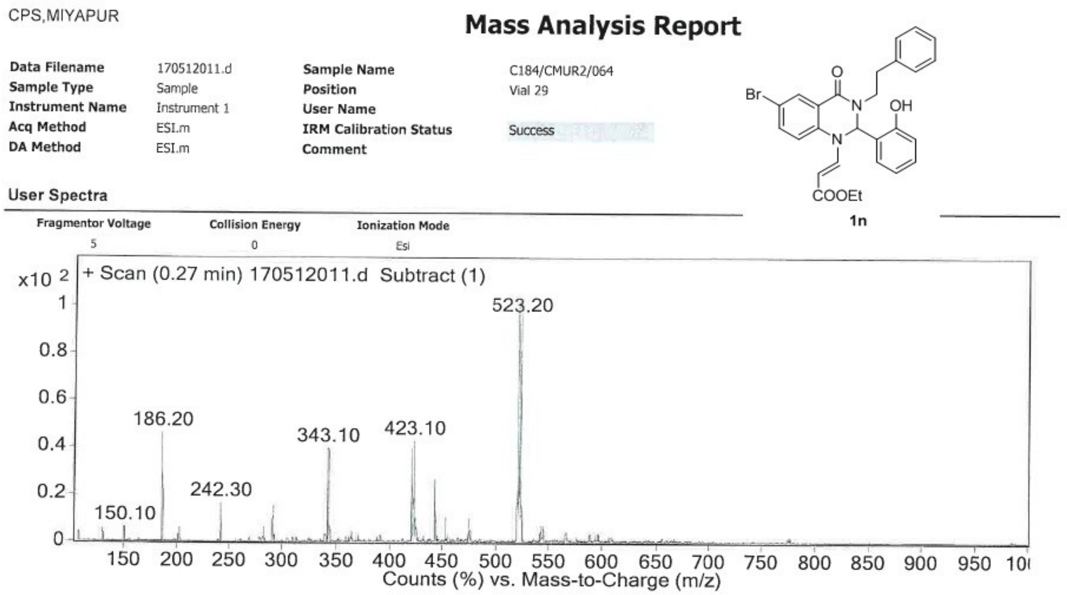

^{1}H NMR of 1o:

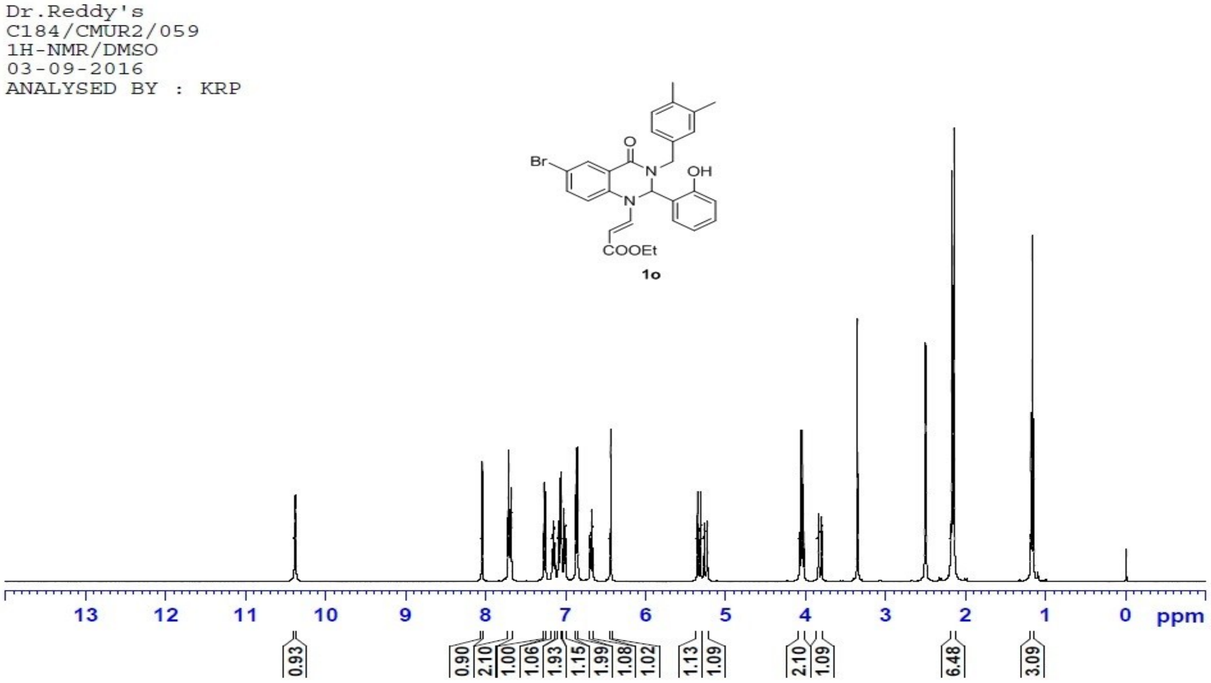

^{13}C NMR of 1o:

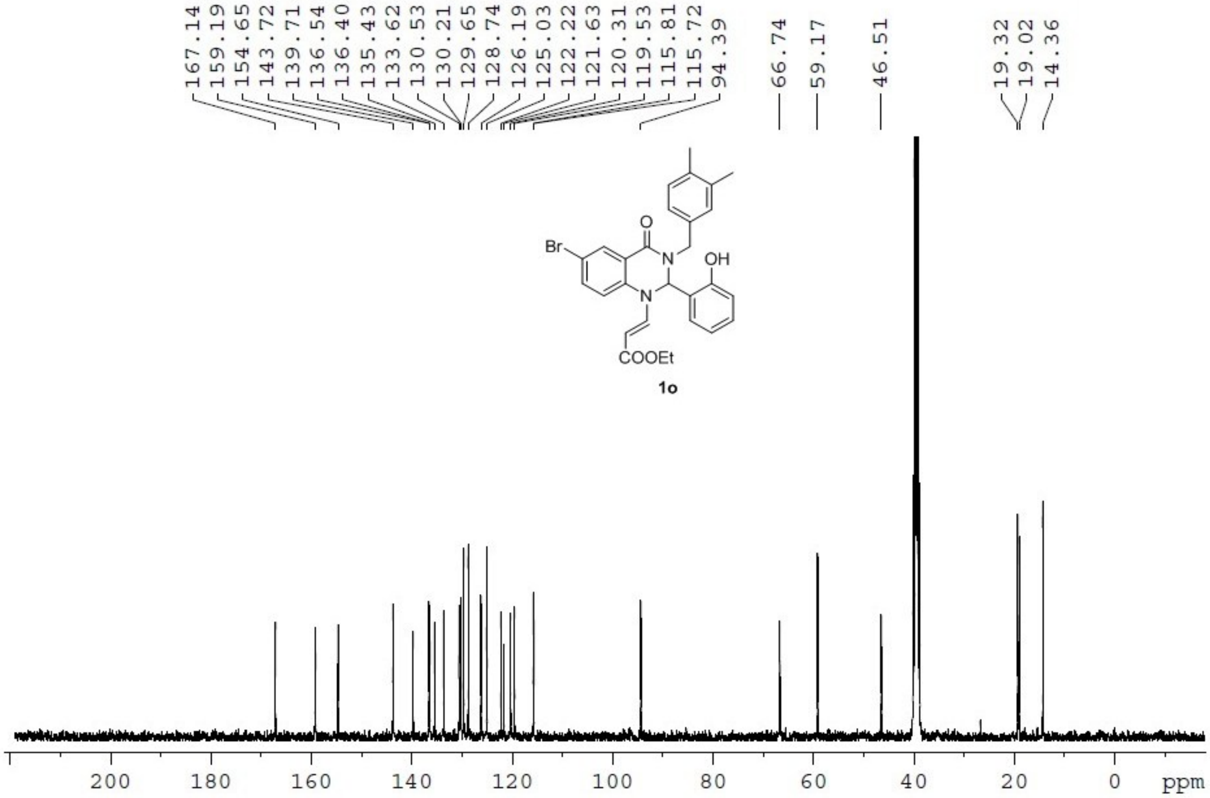

HRMS of 1o:

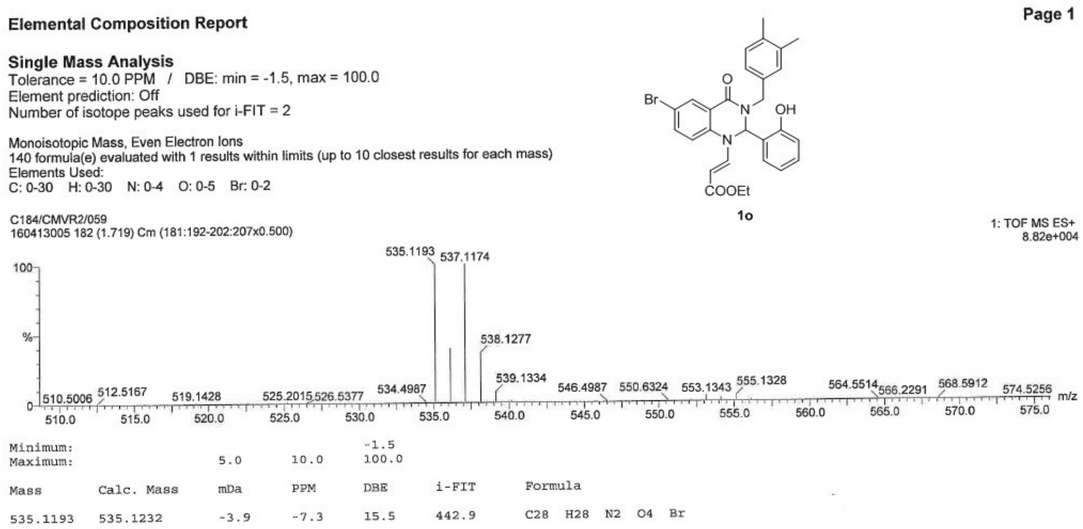

^{1}H NMR of 1p:

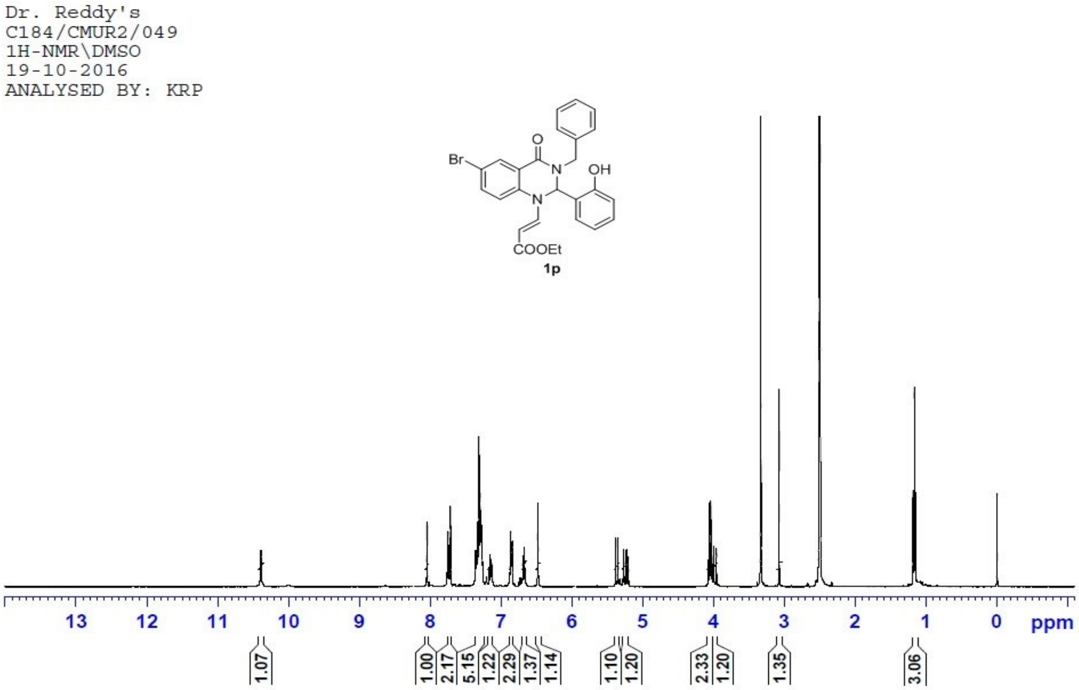

^{13}C NMR of 1p:

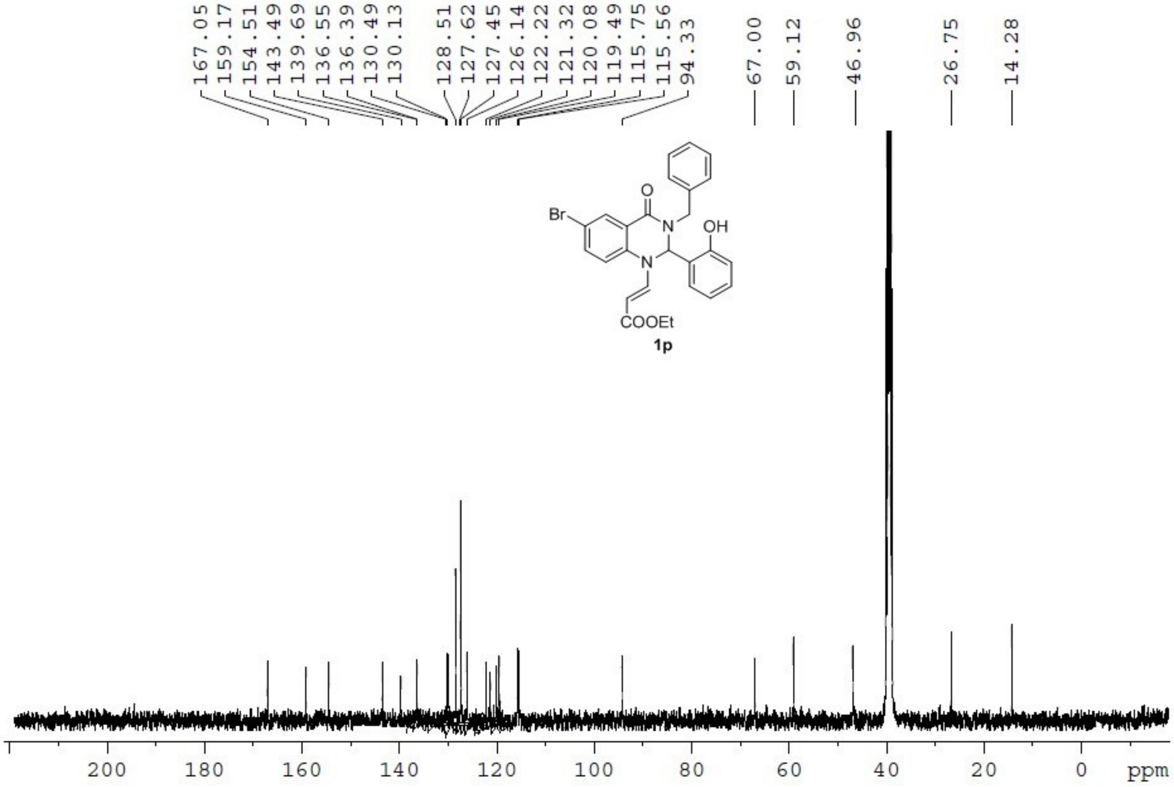

HRMS of 1p:

Elemental Composition Report **Page 1**

Single Mass Analysis
Tolerance = 5.0 PPM / DBE: min = -1.5, max = 50.0
Selected filters: None

Monoisotopic Mass, Even Electron Ions
80 formula(e) evaluated with 1 results within limits (up to 50 best isotopic matches for each mass)
Elements Used:
C: 0-30 H: 0-35 N: 0-3 O: 0-6 Br: 0-1

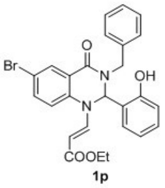

C184/CMUR2/049
161114022 11 (0.251) Cm (8:12)

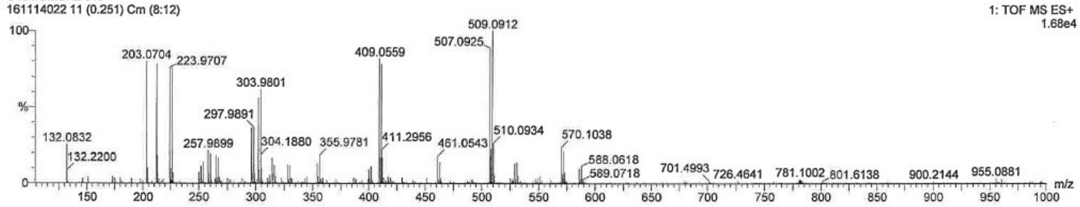

Minimum:				-1.5		
Maximum:		5.0	5.0	50.0		
Mass	Calc. Mass	mDa	PPM	DBE	i-FIT	Formula
507.0925	507.0919	0.6	1.2	15.5	118.6	C26 H24 N2 O4 Br

^{1}H NMR of Intermediate:

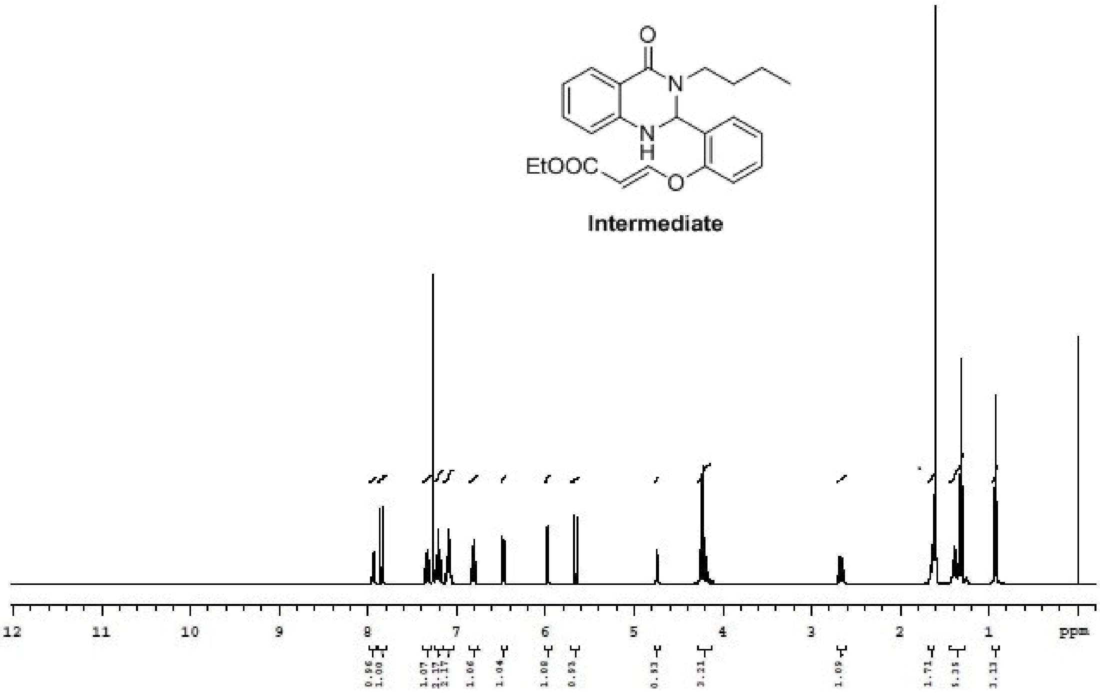

^{13}C NMR of Intermediate:

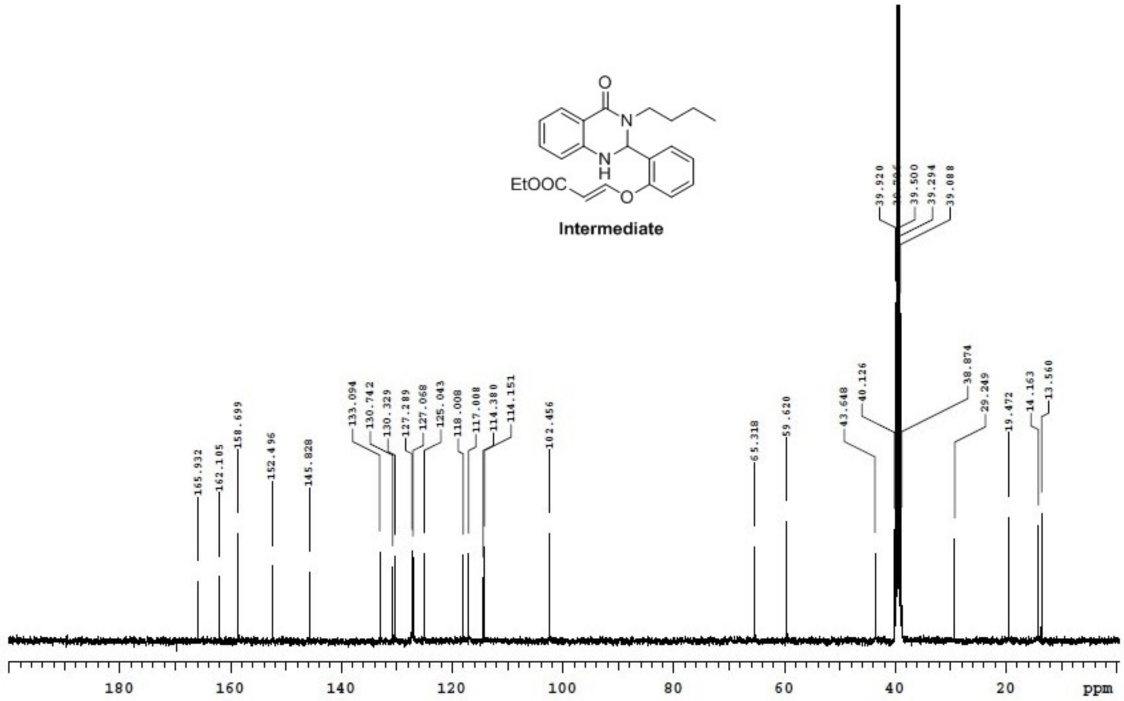

HRMS of Intermediate:

Elemental Composition Report

Page 1

Single Mass Analysis
Tolerance = 5.0 PPM / DBE: min = -1.5, max = 100.0
Element prediction: Off
Number of isotope peaks used for i-FIT = 2

Monoisotopic Mass, Even Electron Ions
75 formula(e) evaluated with 1 results within limits (up to 10 closest results for each mass)
Elements Used:
C: 0-32 H: 0-35 N: 0-3 O: 0-5
C184/CMUR2/043
151222005 31 (0.573) Cm (31:34)

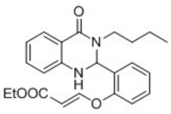

Intermediate

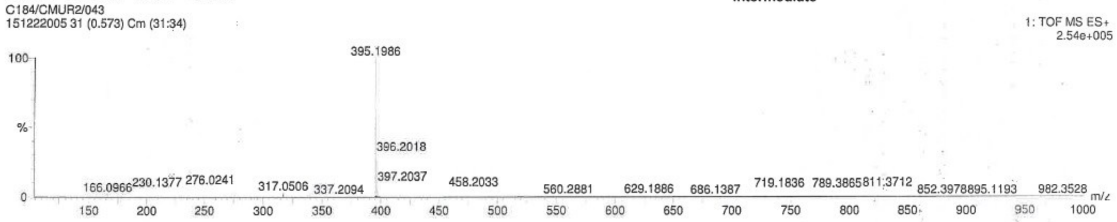

Minimum:				-1.5		
Maximum:		5.0	5.0	100.0		
Mass	Calc. Mass	mDa	PPM	DBE	i-FIT	Formula
395.1986	395.1971	1.5	3.8	11.5	15.4	C23 H27 N2 O4
